# Screening for Asymptomatic Coronary Artery Disease *via* Exercise Stress Testing in Patients With Type 2 Diabetes Mellitus: A Systematic Review and Meta-Analysis

**DOI:** 10.3389/fcvm.2021.770648

**Published:** 2021-11-01

**Authors:** Yaoshan Dun, Shaoping Wu, Ni Cui, Randal J. Thomas, Thomas P. Olson, Nanjiang Zhou, Qiuxia Li, Suixin Liu

**Affiliations:** ^1^Division of Cardiac Rehabilitation, Department of Physical Medicine & Rehabilitation, Xiangya Hospital of Central South University, Changsha, China; ^2^National Clinical Research Centre for Geriatric Disorders, Xiangya Hospital of Central South University, Changsha, China; ^3^Division of Preventive Cardiology, Department of Cardiovascular Medicine, Mayo Clinic, Rochester, MN, United States

**Keywords:** exercise stress testing (EST), coronary artery disease (CAD), type 2 diabetes mellitus(T2DM), diagnostic test, meta-analysis

## Abstract

**Objectives:** This meta-analysis aims to investigate the diagnostic value of exercise stress testing (EST) for asymptomatic coronary artery disease (CAD) among patients with type 2 diabetes mellitus (T2DM) and to ascertain the influence of different variables on the sensitivity and specificity of EST.

**Background:** Asymptomatic CAD occurs in >1 in five diabetes mellitus patients, and it is associated with an increased risk of complications. Methods for screening asymptomatic CAD in T2DM patients are still not unified.

**Methods:** MEDLINE (*via* Ovid), Embase (*via* Ovid), Cochrane Library, SCOPUS, PubMed, Ovid, EBSCO ASP, and Web of Science were systematically searched on June 8 and 9, 2021, for diagnostic cohort and case-control studies. We included studies that used EST to screen for CAD in asymptomatic patients with T2DM, and that used coronary angiography to diagnose CAD and had reported the basic diagnostic indicators. The Quality Assessment of Diagnostic Accuracy Studies 2 tool was used to assess study quality. The combined effect sizes were calculated by overall analysis and multiple variable effects were explored by regression analysis and subgroup analysis.

**Results:** Nine groups of data from eight diagnostic cohort studies, totaling 515 participants, were included. Included studies showed a low risk of bias in most items, except for flow and timing. The combined sensitivity and specificity of EST for asymptomatic CAD in patients with T2DM were 55 (48 to 61%) and 66 (61 to 70%), respectively. When non-diagnostic tests were excluded, sensitivity increased to 73 (56 to 88%). The proportion receiving angiography also significantly affected sensitivity. No significant difference was found in the duration of diabetes or other additional risk factors.

**Conclusions:** EST is a tool of moderate sensitivity and specificity to be used for the initial screening of asymptomatic CAD in T2DM. It has the advantage of being non-invasive, relatively inexpensive, easily available in most settings, and has no radiation associated with its use. Additional research with higher quality studies in which tests that are non-diagnostic are included and flow and timing is described clearly, will be important to further our understanding of EST for asymptomatic CAD detection in patients with T2DM.

**Systematic review registration:** PROSPERO CRD42021259555.

## Introduction

By modern estimates, approximately 425 million (6%) people have diabetes worldwide, with type 2 diabetes mellitus (T2DM) accounting for the majority (>85%) (1). The incidence of asymptomatic coronary artery disease (CAD) in diabetics is between two and seven times higher than in non-diabetic patients (2). Studies (2) have demonstrated that asymptomatic CAD occurs in >1 in five (22%) diabetics, which is associated with autonomic neuropathy involving afferent sympathetic fibers (3, 4). Asymptomatic myocardial ischemia is associated with an increased risk of complications such as myocardial infarction due to delayed diagnosis and treatment (5). Moreover, patients with T2DM present a higher incidence of cardiovascular events and death after a first myocardial infarction (4, 6, 7).

A recent meta-analysis (8) concluded that compared with standard care, non-invasive CAD screening reduced cardiac events by 27% in asymptomatic diabetic patients. In practice, Wackers et al. (2) found that selecting only patients who met the American Diabetes Association guidelines would have failed to identify 41% of patients with silent ischemia. Current recommendations (9) advocate CAD screening in asymptomatic diabetics with high risk. Further research into screening strategies for asymptomatic diabetic patients is warranted.

The methods for screening asymptomatic CAD in diabetics may vary and are not unified. While coronary angiography is the gold standard for identifying CAD, this invasive technique is reserved for patients with evidence of ischemia on a stress test or for those with continuous cardiac symptoms (10). Therefore several non-invasive tools have been recommended for primary screening of asymptomatic CAD in diabetics, including exercise stress testing (EST), single-photon emission computed tomography (SPECT), multidetector computed tomography (MDCT), coronary computed tomography angiogram (CCTA), and stress echocardiography (11).

Among those approaches, EST is the most common tool applied to individuals with suspected CAD (12). Compared with alternative methods, EST is non-invasive, cost-effective, free from radiation, and widely available, affirming its appropriateness as an initial screening tool. In addition, it also has prognostic value by providing information on exercise capacity, dysrhythmia evaluation, heart rate response and hemodynamic response (13).

Gianrossi et al. (14) observed high sensitivity and specificity of EST for CAD in the general population, but with wide variability (mean sensitivity, 68%; range, 23–100%; and mean specificity, 77%; range, 17–100%). While the diagnostic values of exercise electrocardiograph (ECG) testing in diabetics was first explored in a review (15), no combined diagnostic values of EST were calculated based on the small size and higher verification bias of included studies. To date, there is no higher-level clinical evidence to probe into the diagnostic value of EST in asymptomatic patients with T2DM. Moreover, due to the wide variability of diagnostic values across different studies, we lack a systematic understanding of how various factors contribute to the sensitivity and specificity of EST detecting asymptomatic CAD for diabetics.

Therefore, the purpose of this meta-analysis is to investigate the diagnostic value of EST for detecting asymptomatic CAD patients with T2DM; and to ascertain the influence of different variables (population, technical and methodologic factors) on the sensitivity and specificity of EST for asymptomatic CAD detection of T2DM patients.

## Methods

The methods and results of this meta-analysis are presented according to the Preferred Reporting Items for Systematic reviews and Meta-Analysis statement (PRISMA) (Supplementary PRISMA DTA Checklist). The meta-analysis was registered at PROSPERO (CRD42021259555).

### Criteria for Screening Studies

#### Inclusion Criteria

Articles were included if they fulfilled the following criteria; (a) cohort or case-control studies; (b) participants diagnosed with T2DM but without any coronary disease (e.g., stable angina, unstable angina, myocardial infarction) confirmed prior to participation; (c) EST must be used to screen CAD in T2DM patients on a bicycle ergometer or treadmill with a 12-lead ECG recorded during testing, with invasive coronary angiography as the gold standard; (d) the outcome data can be derived, including true positive (TP), false positive (FP), false negative (FN), and true negative (TN).

#### Exclusion Criteria

Studies were excluded if the full-text was unavailable, participants were only type 1 diabetes mellitus, the EST detection criteria was not ST depression, such as exercise capacity, heart rate response, or a 2 × 2 diagnostic table could not be reconstructed.

### Search Methods

MEDLINE, Embase, SCOPUS, PubMed, Ovid, EBSCO ASP, and Web of Science were searched by using a strategy combining selected Medical Subject Headings (MeSH) terms (Exercise test; Diabetes Mellitus, Type 2; Coronary Artery Disease, Myocardial Ischemia, Heart disease) and free-text terms. Additionally, we searched ClinicalTrial.gov to determine whether there were related clinical trials being carried out. The search term regarding diagnostic study design (sensitiv^*^ OR [sensitivity and specificity] OR [predictive AND value^*^] OR predictive value of tests OR accuracy^*^), was obtained through the website of McMaster university health information. We imposed no language or other limitations. The detailed search strategies are listed in Supplementary Methods. The last search was performed on June 9, 2021.

**Figure 1:**
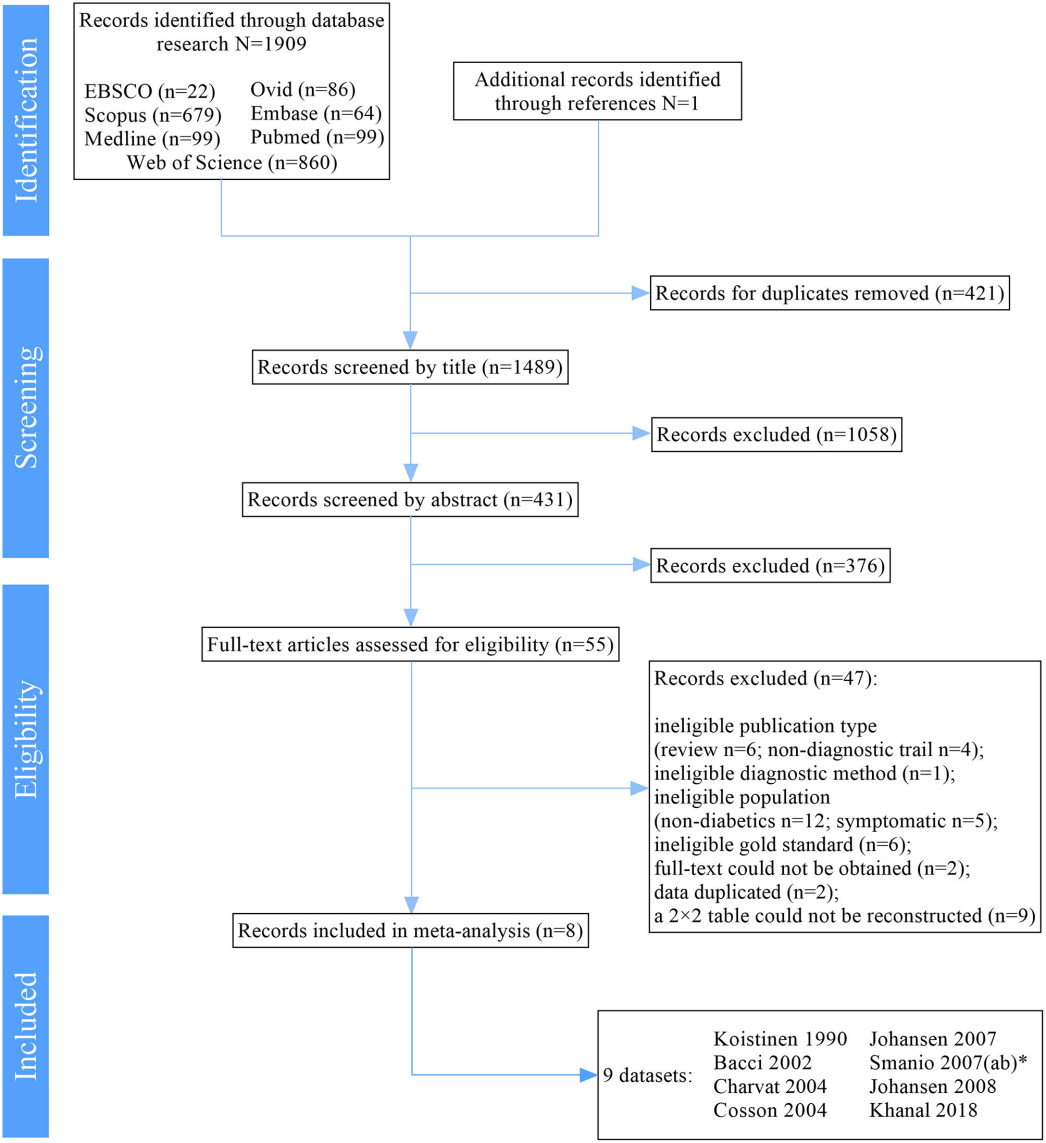
PRISMA Flow Diagram. PRISMA: Preferred Reporting Items for Systematic reviews and Meta-Analysis statement. *ab represents 2 different exercise protocols of EST for detection of CAD in one study (16).

Information regarding the inclusion/exclusion of studies is summarized in Figure 1.

### Data Collection

Two authors (NC and SW) independently screened each record retrieved from the search after deduplication. The full-text reports of all potentially relevant diagnostic studies were obtained and independently assessed for eligibility based on the defined inclusion criteria. Thereafter, participant characteristics, outcomes, technical and methodologic factors of the included studies were extracted using a standardized data collection form which had been piloted on two records included in the meta-analysis.

Any disagreement during the process was resolved through discussion, and where uncertainty remained, two additional authors (YD and SL) were consulted for consensus.

### Assessment of Methodologic Quality

We used the Quality Assessment of Diagnostic Accuracy Studies 2 (QUADAS-2) tool to assess the quality of included studies (17). Two authors independently evaluated the risk of bias and the applicability concerns, and if there were discrepancies, these were resolved *via* discussion or reviewed by other authors.

### Overall Analysis

Heterogeneity amongst included studies was explored from both diagnostic and non-diagnostic thresholds in Meta-DiSc 1.4 (18) and STATA 15.1 (StataCorp LLC, US). Where appropriate, the results from included studies were combined for each outcome to give an overall estimate of diagnostic effect. A fixed-effect meta-analysis would be used if *I*^2^ ≤ 50%, if not, a random-effects model would be used.

The Spike plot was used for sensitivity analysis to check for particularly influential observations using Cook's distance. The Deek's funnel plot asymmetry test was used to examine publication bias for outcomes.

### Univariate Regression Analysis

To explore the origin of heterogeneity, we carried out regression analysis in JMP Pro 14 (SAS, NC, USA). Given the relatively small ratio of trials to covariates, multivariable meta-regression was not appropriate, and instead, limited to a univariate analysis. Subsequently, we carried out subgroup analysis for further exploration of statistically significant items. Regarding missing data, we made a chart to present the detailed percentage we collected, and we would discard those missing more than 60% of the data. All collected information is displayed in Table 1.

**Table 1:** Variables abstracted from exercise stress testing literature.

**Population characteristics (15 variables)**	**Technical factors (12 variables)**	**Methodologic factors (5 variables)**
Mean age	Publication year	Was the exercise ECG being compared with a better test? (yes/no)
Percent men	Continent of study center	
Mean duration of diabetes (years)	Exercise protocol (treadmill, bicycle)	Whether all participants were included? (yes/no)
Were patients with these conditions excluded from the study? (yes/no)	Smallest amount of ST depression deemed abnormal (1, > 1, 1.5 mm)	
		Did the authors comply with these standards? (yes/no)
Left ventricular hypertrophy	Point in time when measurement was made ST depressions adjusted for heart rate? (yes/no)	
Right bundle branch block		Blind reading of angiogram
Left bundle branch block		Blind reading of ECG
Mitral valve prolapse	Computer algorithm used to analyze ST	Treatment of equivocal or non-diagnostic test were
Resting repolarization abnormalities?	Segment? (yes/no)	
	Percent patients achieving “adequate heart rate”	Excluded from analysis
Whether all patients were T2DM?		Included and considered as normal tests
Percent of the study group with	Mean workload achieved (W)	Not mention about these patients
Hypertension	Mean heart rate achieved (bpm)	
Smoking	Mean double product achieved	
Lipid abnormalities	Time interval between exercise test and coronary angiogram	
Family history of CAD		
Were patients taking these medications excluded from the study? (yes/no)	Angiographic definition of disease (50% vs. others)	
P-receptor-blocking agents		
Long-acting nitrates		

*CAD, Coronary heart disease; ECG, Electrocardiograp; T2DM, Type 2 diabetes mellitus*.

### Subgroup Analysis

Subgroup analysis was performed in STATA 15.1 to examine potential diagnostic effect modifiers. We tested the following a priori hypotheses that there may be differences in the diagnostic effect of EST on sensitivity and specificity:

- Type of diabetes (mixed: type 1 and 2; or only type 2);
- Exercise protocol (treadmill or bicycle ergometer);
- The proportion of included participants (all-included or proportion-included);
- Angiographic criteria of CAD (50% or others).

## Results

### Description of Studies

#### Results of the Search

We traced 1,909 results from multiple electronic sources. After removal of duplicates, title and abstract screening, 55 records remained. Then, 47 records were excluded after the full-text screening. Finally, eight (16, 19–25) studies were included in our meta-analysis and systematic review. This selection process is summarized in Figure 1.

#### Included Studies

All of the eight studies (nine datasets) were diagnostic cohort studies and included 515 asymptomatic diabetics. The sample size of most studies was relatively small (median 64 participants, range: 28–104). Among 515 patients, 177 diabetics (34%) were diagnosed with CAD by angiography (range 27–51% in a single study). The average age of participants in the trials ranged from 48 to 60 years, and the mean duration of diabetes ranged from 6.0 to 12.9 years. Moreover, many participants had co-existing cardiovascular risk factors apart from diabetes, 67% had hypertension (range: 28–100%), 34% with smoking history (range: 17–65%), 67% with lipid abnormalities (rang: 45–89%) and 35% with a family history of CAD (range: 5–63%). One study (16) only included women. Men accounted for 51% of the total included participants. Two studies (19, 22) included a mixed population with type 1 and type 2 diabetes and the remaining six studies included only type 2 diabetics.

In two studies (20, 21), the angiographic definition of CAD was a narrowing of 70% or greater in the cross-sectional area of one coronary artery. One study (22) defined CAD as a ≥70% narrowing of the coronary artery, or ≥50% diameter narrowing of the left main coronary artery, while the remaining studies defined CAD as a 50% narrowing. In five studies (19–22, 25), participants were screened by two or more non-invasive screenings, including EST and other tools such as SPECT or MDCT or stress echocardiography. When at least one of these non-invasive tests was positive, angiography would be conducted and only patients who received both EST and angiography were included in our meta-analysis. While this may contribute to ascertainment bias, those studies were included due to the small number of relevant studies available. We had performed subgroup analysis (the proportion of included participants) to assess the impact of bias. EST indication in four studies (19, 20, 23, 24) was an ST depression ≥1 mm persisting for at least 0.08s after the J point, and in two studies (21, 22) was an ST depression >1 mm and in one study was an ST depression ≥1.5 mm (16). Five studies (19, 21–24) used cycle ergometry and the others used treadmill ergometry. Only one study (19) reported no adverse events during EST.

All studies reported diagnostic values available for a 2 ×2 table reconstruction. Details of included studies are listed in Table 2.

**Table 2:** Characteristics of included studies.

**References**	**Study design**	**Sample size (*n*)**	**Population constitution**	**Clinical presentation**	**Exercise protocol**	**EST indication of CAD**	**Reference standard used**	**Angiographic criteria of CAD**
Koistinen et al. (19)	Cohort	33	Type 1 and type 2 diabetics	Asymptomatic	Bicycle	ST depression ≥1 mm and persisted for at least 0.08 s after the J point	Coronary angiogram	≥50% narrowing
Bacci et al. (20)	Cohort	71	Type 2 diabetics	Asymptomatic	Treadmill	ST depression ≥1 mm and persisted for at least 0.08 s after the J point	Coronary angiogram	≥70% narrowing
Charvat et al. (21)	Cohort	30	Type 2 diabetics	Asymptomatic	Bicycle	ST depression >1 mm and persisted for at least 0.08 s after the J point	Coronary angiogram	≥70% narrowing
Cosson et al. (22)	Cohort	76	Type 1 and type 2 diabetics	Asymptomatic	Bicycle	ST depression >1 mm and persisted for at least 0.08 s after the J point	Coronary angiogram	≥50% or ≥70% narrowing*
Johansen et al. (23)	Cohort	82	Type 2 diabetics	Asymptomatic	Bicycle	ST depression ≥1 mm	Coronary angiogram	≥50% narrowing
Smanio et al. (16)†	Cohort	104	Type 2 diabetics	Asymptomatic	Treadmill	ST depression ≥1.5 mm in relation to baseline or exercise-induced ischemia	Coronary angiogram	≥50% narrowing
Smanio et al. (16)†	Cohort	104	Type 2 diabetics	Asymptomatic	Bicycle	ST depression ≥1.5 mm in relation to baseline or exercise-induced ischemia	Coronary angiogram	≥50% narrowing
Johansen et al. (24)	Cohort	91	Type 2 diabetics	Asymptomatic	Bicycle	ST depression ≥1 mm	Coronary angiogram	≥50% narrowing
Khanal et al. (25)	Cohort	28	Type 2 diabetics	Asymptomatic	Treadmill	Exercise ECG	Coronary angiogram	≥50% narrowing

*CAD, Coronary heart disease; ECG, Electrocardiograph. EST, Exercise stress testing.*

^*^≥50% for the left main coronary artery and ≥70% for others coronary artery.

*Different exercise protocols of EST for detection of CAD in one study (16)*.

#### Risk of Bias in Included Studies

Details on the methodologic quality of included studies are available in Supplementary Figures 1, 2. Only one study (23) showed a low risk of bias in all items, and five studies (19–22, 25) reported a high risk in flow and timing.

While two studies reported low risk in patient selection, five studies showed unclear risk. Two studies (20, 23) included consecutive or randomized patients, while one (16) recruited patients *via* phone-call or e-mail. All included studies avoided inappropriate exclusions except for one study (24) which did not report exclusion criteria of participants.

Most studies reported low risk in the index test, except one study (16) reported unclear risk. Reference test was performed before index test in one study (16), without description blinding assessment of index test.

The details that the reference standard results interpreted without knowledge of the index test results were only described in four studies. In general, four studies (20–22, 25) were judged as unclear risks of standard reference bias.

A high risk of bias was observed in the flow and timing. Information about the interval between index tests and the reference standard was not described in four included studies (21, 22, 24, 25). Five studies (19–22, 25) were judged as high risk of bias without appropriate analysis of all included patients.

### Diagnostic Performance of EST

#### Overall Analysis

The data of the overall meta-analysis are provided in Supplementary Table 1: 2 ×2 table.

The Spearman correlation coefficient was 0.21 (*p* = 0.59 > 0.05), showing no significant threshold effect in this study. Furthermore, the symmetric SROC curve (Supplementary Figure 3) was drawn without “shoulder and arm shape,” which further demonstrates no threshold effect.

The result of the Cochran-Q test for DOR indicated heterogeneity caused by the non-threshold effect exists in included studies (Cochran-Q = 25.98, *p* < 0.01). Furthermore, *I*^2^ of sensitivity, specificity, positive likelihood ratio (LR^+^), negative likelihood ratio (LR^−^), and DOR were all significantly high. A random-effect model was used to estimate the five effect sizes above, which might only serve as a reference on account of its high heterogeneity.

Based on the nine datasets, the combined sensitivity and specificity of EST were 55 (48 to 61%) and 66 (61 to 70%), respectively (Supplementary Figure 4). Combined LR^+^ of EST was 1.52 (1.08 to 2.13), combined LR^−^ was 0.74 (0.55 to 0.99), the combined area under the curve (AUC) was 0.66, combined Q index was 0.62, and combined DOR was 2.33 (1.17 to 4.65). Besides, the combined positive predictive value was 47 (34 to 59%), and the combined negative predictive value was 74 (68 to 80%) (Supplementary Figure 5).

Supplementary Figure 6 demonstrates the result of sensitivity analysis. The sensitivity of all the original studies is low suggesting the results of this study are relatively stable.

We found no publication bias in the regression test for funnel plot asymmetry (Deek's test *p* = 0.25>0.05; Supplementary Figure 7).

#### Univariate Regression Analysis

Supplementary Figure 8 shows the percentage of missing data for those items in Table 1. Two variables (mean double product achieved and mean workload achieved) were excluded in univariate regressions analysis because more than 60% of the data were missing for these variables. One variable (point in time when the measurement was made) was excluded as it was the same in all studies.

Table 3 displays the results of the univariate regressions analysis. Studies that included patients who had partially received angiography calculated significantly higher sensitivity than those all received (*p* < 0.001). Also, studies that excluded non-diagnostic tests from analysis reported significantly higher sensitivity than studies that did not mention these patients (*p* = 0.03). Other variables showed no significant relationship with both sensitivity and specificity in the analysis.

**Table 3:** Variables associated with sensitivity and specificity by univariate regression analysis.

**Variables**	**Sensitivity coefficient [95%CI]**	***p***	**Specificity coefficient [95%CI]**	***p***
Mean age	−0.02 [−0.08 to 0.03]	0.33	−0.01 [−0.06 to 0.04]	0.75
Percent men	0.107 [−0.56 to 0.78]	0.72	−0.003 [−0.50 to 0.49]	0.99
Mean duration of diabetes (years)	0.05 [−0.03 to 0.13]	0.17	−0.02 [−0.10 to 0.05]	0.42
Left ventricular hypertrophy	−0.08 [−0.36 to 0.20]	0.5	0.16 [−0.004 to 0.32]	0.06
Right bundle branch block	−0.08 [−0.36 to 0.20]	0.5	0.16 [−0.004 to 0.32]	0.06
Left bundle branch block	−0.09 [−0.29 to 0.12]	0.34	0.02 [−0.14 to 0.18]	0.76
Mitral valve prolapse	−0.08 [−0.36 to 0.20]	0.5	0.16 [−0.004 to 0.32]	0.06
Resting repolarization abnormalities?	−0.03 [−0.24 to 0.19]	0.79	0.01 [−0.16 to 0.17]	0.94
only type 2 or mixed type 1/2	−0.07 [−0.28 to 0.14]	0.46	0.05 [−0.10 to 0.20]	0.47
Hypertension	0.16 [−0.67 to 1.00]	0.64	−0.05 [−0.84 to 0.73]	0.87
smoking	−0.43 [−1.80 to 0.95]	0.46	0.26 [−1.05 to 1.57]	0.63
Lipid abnormalities	−0.46 [−4.11 to 3.18]	0.64	0.76 [−1.11 to 2.63]	0.22
Family history of CAD	−0.48 [−1.25 to 0.30]	0.17	0.48 [−0.20 to 1.16]	0.13
p-receptor-blocking agents	0.09 [−0.11 to 0.30]	0.33	−0.03 [−0.19 to 0.13]	0.7
long-acting nitrates	0.09 [−0.11 to 0.30]	0.33	−0.03 [−0.19 to 0.13]	0.7
Publication year	−0.01 [−0.04 to 0.01]	0.37	−0.01 [−0.03 to 0.01]	0.17
Continent of study center*		0.57		0.18
Exercise protocol (treadmill, bicycle)	0.001 [−0.18 to 0.18]	0.99	−0.004 [−0.14 to 0.13]	0.94
Smallest amount of ST depression deemed abnormal (1, >1, 1.5 mm)*		0.57		0.11
ST depressions adjusted for heart rate (yes/no)	0.16 [−0.10 to 0.41]	0.18	−0.03 [−0.24 to 0.18]	0.75
Computer algorithm used to analyze ST segment	0.16 [−0.1 to 0.41]	0.18	−0.03 [−0.24 to 0.18]	0.75
Percent patients achieving “adequate heart rate”	1.33 [−8.00 to 10.65]	0.6	2.33 [−6.98 to 11.64]	0.39
Mean heart rate achieved (bpm)	0.02 [−0.01 to 0.06]	0.09	0.001 [−0.04 to 0.04]	0.94
Time interval between exercise test and coronary angiogram	−0.05 [−0.24 to 0.15]	0.42	−0.002 [−0.14 to 0.14]	0.96
Angiographic definition of disease (50% vs. others)	−0.09 [−0.27 to 0.08]	0.24	0.01 [−0.14 to 0.15]	0.93
Was the exercise ECG being compared with a “better” test (yes/no)	−0.04 [−0.22 to 0.14]	0.64	0.05 [−0.08 to 0.17]	0.43
Whether all participants were included? (yes/no)	0.18 [0.09 to 0.27]	0.002	−0.05 [−0.18 to 0.07]	0.37
Blind reading of angiogram	0.12 [−0.03 to 0.27]	0.1	−0.07 [−0.19 to 0.05]	0.22
Blind reading of ECG	−0.04 [−0.26 to 0.17]	0.66	−0.07 [−0.22 to 0.07]	0.28
Treatment of equivocal or non-diagnostic test were	0.15 [0.02 to 0.28]	0.03	0.01 [−0.12 to 0.15]	0.85

*p-value among the two factors in this item was calculated.

#### Subgroup Analysis

**Figure 2:**
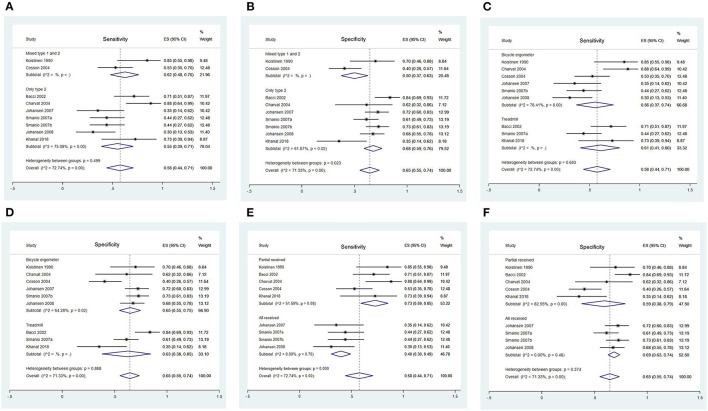
Forest Plots of Sensitivity and Specificity of EST in Different Subgroups. **(A)** Forest plot of sensitivity of EST in different type of diabetes; **(B)** Forest plot of specificity of EST in different type of diabetes; **(C)** Forest plot of sensitivity of EST in different exercise protocol; **(D)** Forest plot of specificity of EST in different exercise protocol; **(E)** Forest plot of sensitivity of EST in different proportion of included participants; **(F)** Forest plot of specificity of EST in different proportion of included participants.

**Figure 3:**
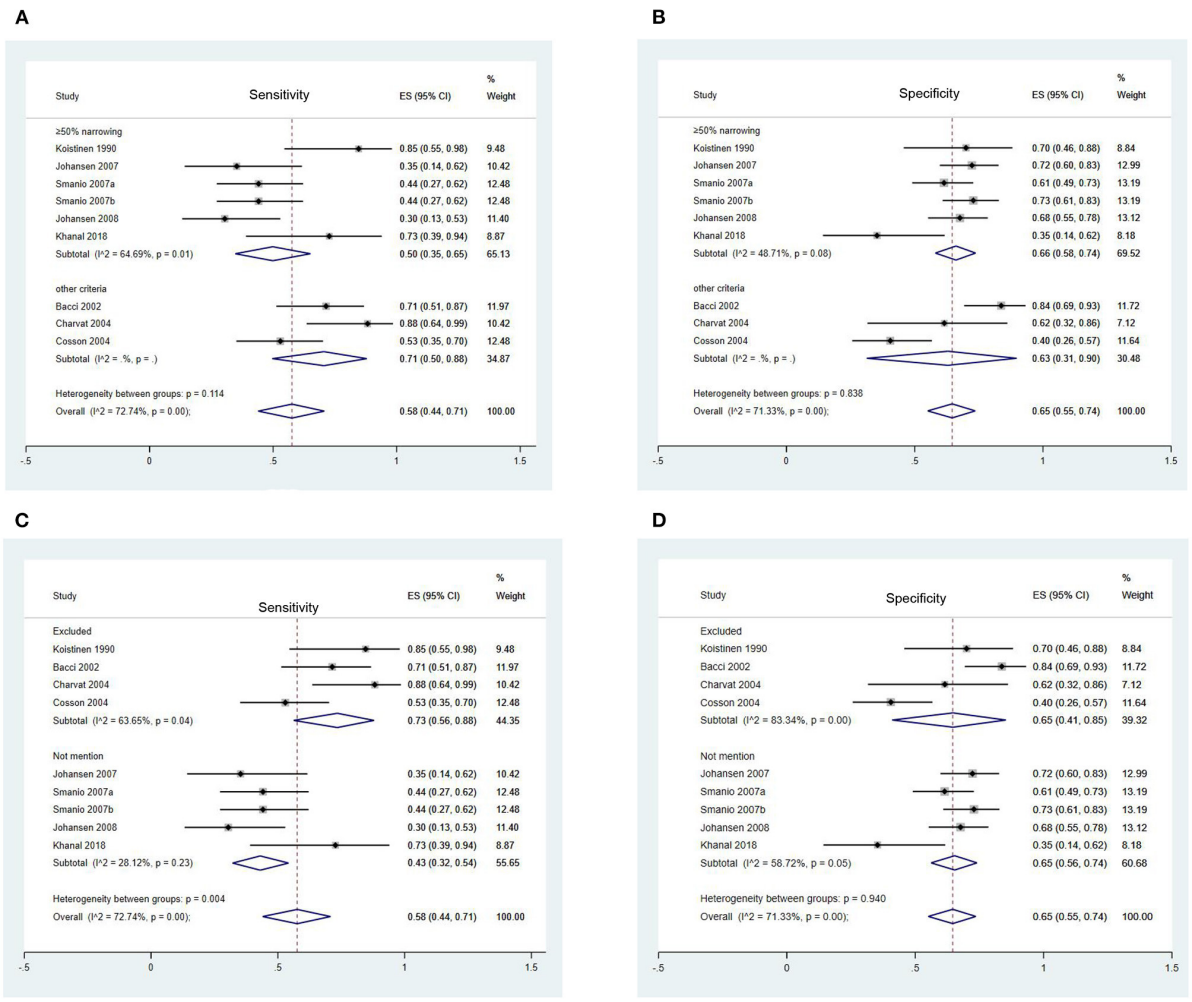
Forest Plots of Sensitivity and Specificity of EST in Different Subgroups. **(A)** Forest plot of sensitivity of EST in different angiographic criteria of CAD; **(B)** Forest plot of specificity of EST in different angiographic criteria of CAD; **(C)** Forest plot of sensitivity of EST in different treatment equivocal or non-diagnostic test; **(D)** Forest plot of specificity of EST in different treatment equivocal or non-diagnostic test.

Forest Plots of Sensitivity and Specificity of EST for CAD detection in different subgroups are presented in Figures 2, 3. Figure 2 shows the sensitivity and specificity of different types of diabetes (Figures 2A,B), different exercise protocol (Figures 2C,D), and different proportions of patients included from studies (Figures 2E,F). Figure 3 shows the sensitivity and specificity of different angiographic criteria of CAD (Figures 3A,B) and different treatment equivocal or non-diagnostic tests (Figures 3C,D).

#### Type of Diabetes

No significant heterogeneity was observed in sensitivity between two subgroups (*p* = 0.50) (Figure 2A), while it existed in specificity (*p* = 0.02) (Figure 2B). It might indicate that the specificity of EST in mixed type 1 and 2 diabetics [50 (37 to 63%)] was significantly lower than only type 2 diabetics population [68 (59 to 76%)].

#### Exercise Protocol

No significant heterogeneity was observed in sensitivity and specificity between two subgroups (sensitivity: *p* = 0.69; specificity: *p* = 0.87) (Figures 2C,D).

#### The Proportion of Included Participants

Significant heterogeneity was observed in sensitivity between two groups (*p* < 0.001) (Figure 2E). Studies where only a proportion of participants were included presented a higher sensitivity [73 (59 to 85%)] than those in which all participants included [40 (30 to 49)]. No significant heterogeneity was observed in specificity between two subgroups (*p* = 0.37) (Figure 2F).

#### Angiographic Criteria of CAD

No significant heterogeneity was observed in both sensitivity (*p* = 0.11) and specificity (*p* = 0.84) between two subgroups (Figures 3A,B).

#### Treatment of Equivocal or Non-diagnostic Test

Significant heterogeneity was shown in sensitivity between two groups (*p* = 0.004) (Figure 3C). Sensitivity in studies that excluded these patients [73 (56 to 88%)] from the analysis was significantly higher than those without mention [43 (32 to 54%)]. No significant heterogeneity was observed in specificity (*p* = 0.84) between two subgroups (Figure 3D).

The sensitivity and specificity of EST for CAD according to total population and subgroups are listed in Table 4.

**Table 4:** Sensitivity and specificity of EST for coronary artery disease according to total population and subgroups.

	**Diagnostic performance estimate**			
	**Sensitivity**		**Specificity**	
	**Estimate**	**95% CI**	**Estimate**	**95% CI**
**Total**	0.58	0.44–0.71	0.65	0.55–0.74
**Type of diabetes**				
Mixed type 1 and 2	0.62	0.48–0.76	0.50*	0.37–0.63
Only type 2	0.55	0.39–0.71	0.68*	0.69–0.76
**Exercise protocol**				
Bicycle ergometer	0.56	0.37–0.74	0.65	0.55–0.75
Treadmill	0.61	0.41–0.80	0.63	0.38–0.85
**The proportion of included participants** ^†^				
All included	0.40*	0.30–0.49	0.69	0.63–0.74
Proportional included	0.73*	0.59–0.85	0.59	0.38–0.79
**Angiographic criteria of CAD**				
Other criteria	0.71	0.50–0.88	0.63	0.31–0.90
≥50% narrowing	0.5	0.35–0.65	0.66	0.58–0.74
**Treatment of equivocal or non-diagnostic test**				
Excluded	0.73*	0.56–0.88	0.65	0.41–0.85
Not mention	0.43*	0.32–0.54	0.65	0.56–0.74

*p ≤ 0.05 in heterogeneity test between groups.

*Only participants who received both EST and angiography were included into our analysis. This is the proportion of participants we include in the original studies*.

## Discussion

To our knowledge, this is the first systematic meta-analysis to investigate the EST screening programme for asymptomatic CAD in T2DM. The results of which suggest that EST is a tool with moderate sensitivity and specificity in the initial screening of asymptomatic CAD in T2DM. It is particularly appealing compared to other screening tool options, since it is non-invasive, relatively inexpensive, easily available in most centers, and involves no radiation.

The present study suggested that studies with proportional participants included had significantly higher sensitivity than those in which all participants were included, suggesting an influence of ascertainment bias in those results. Still, the use of angiography in patients with an abnormal EST may be a cost-effective and clinically feasible approach. We also found that the specificity of EST in the group of mixed type 1 and 2 diabetics were significantly lower than that of only type 2 diabetics in subgroup analysis. This suggests that EST is relatively accurate in identifying T2DM patients without asymptomatic CAD possibly due to patients with T1DM generally developing the disease at a younger age than those with T2DM, as EST has been demonstrated a relatively lower specificity in the youth population (26). Additionally, if the non-diagnostic tests were excluded, the sensitivity of EST would increase substantially from 55 to 73%. Non-diagnostic was defined as “the patient interrupted the test before they reached a heart rate corresponding to 85% of the maximal aerobic capacity without ischemic changes in ECG.” As for the “not mentioned” group, where they did not clarify how non-diagnostic tests were identified, it is not clear whether non-diagnostic tests were included and considered as normal EST screening tests. This may have contributed to why the sensitivity in studies that excluded the non-diagnostic patients (73%) from the analysis was significantly higher than that in studies that did not mention the disposition of non-diagnostic tests (43%). These findings highlight the critical importance of closely following standardized methodology when conducting and interpreting EST in clinical practice. Standardized guidelines (27, 28) have been published detailing specific absolute and relative EST termination and interpretation criteria. In the event that an EST is terminated prior to meeting predefined standardized criteria, that test should be defined as non-diagnostic and data should be interpreted with caution.

Contrary to our expectations, the univariate regression analysis did not find a significant difference regarding the mean duration of diabetes and left bundle branch block. This may be due to the small heterogeneity in diabetes duration of the nine datasets (five articles' mean duration of diabetes = 6 years), or the big difference in article numbers between two subgroups (left bundle branch block: exclude to include = 1 to 8). However, this also indicates that the screening effects of EST are stable, and it would not be interfered with by the above factors.Furthermore, EST in the clinical setting may be achieved with relative safety, a 18-year cross-sectional study from our team, which included 50,142 consecutive tests, suggested that EST is safe with a low rate of adverse events at 0.6 per 10,000 tests (0.2–1.8) (29).

In clinical practice, there are several methods used to assess CAD in asymptomatic diabetic patients. A cost-effectiveness study (30) previously recommended that applying a low-cost test to a large-scale population with selective use of more expensive testing at a later stage for patients with a higher probability of suffering disease is more cost-effective than applying the more expensive test as the initial step. The major advantage of EST is its lower cost compared with most of other methods. Besides, it is readily available and free from radiation, which supports its use as an initial test. Especially when combining clinical information with EST data, they could yielded a 94% sensitivity and 92% specificity (31). Meanwhile, it should be acknowledged that physical disability and vascular and neuropathic changes would make it difficult to reach the target heart rate in EST, which may limit the ability of some patients to complete an EST. Generally, more higher quality studies are needed, in which non-diagnostic tests are excluded and the flow and timing is described clearly.

### Limitations

It is important to recognize potential limitations regarding this meta-analysis. First, over half of the selected articles included only proportional participants from the original studies. Only participants with positive EST or other non-invasive exams would be lead to further gold standard examination, which might increase bias as these included participants might not well-represent this population. To investigate the effect of this bias on the results, we performed a subgroup analysis and offered a detailed explanation. Second, missing data in our subgroup analysis resulted in decreased power of the outcome. Third, these diagnostic studies are often based on preselected populations. The need for exercise ability may limit some patients, which may result in selection bias. Last, most websites we searched have only English reports even though we did not set any limitations in languages and consequently, we may have missed data from essential studies published in other languages.

## Conclusions

EST is a tool with moderate sensitivity and specificity in the initial screening of asymptomatic CAD in T2DM. It is appealing, compared to other screening tools, because it is non-invasive, relatively inexpensive, easily available in most centers, and does not involve radiation. Additional higher-quality studies, where non-diagnostic tests are excluded and the flow and timing are described clearly, are needed to study the use of EST for screening for CAD in T2DM patients.

## Data Availability Statement

The raw data supporting the conclusions of this article will be made available by the authors, without undue reservation.

## Ethics Statement

The studies involving human participants were reviewed and approved by the Ethics Committee of Xiangya Hospital of Central South University.

## Author Contributions

YD contributed to the design, acquisition of data, analysis, funding acquisition and writing (review and editing). SW contributed to the acquisition of data, analysis and writing (original draft). NC contributed to the acquisition of data, analysis and writing (original draft). RT and TO contributed to conceptualization, writing (review and editing). NZ and QL contributed to conceptualization and writing (review). SL contributed to conceptualization, supervision, and funding acquisition. All authors contributed to the article and approved the submitted version, and accept full responsibility for the work and conduct of this study.

## Funding

Grants support were received from the National Natural Science Foundation of China (82002403), Hunan Provincial Natural Science Foundation of China (2021JJ40981), and the Youth Science Foundation of Xiangya Hospital (2019Q03) to YD.

## Conflict of Interest

The authors declare that the research was conducted in the absence of any commercial or financial relationships that could be construed as a potential conflict of interest.

## Publisher's Note

All claims expressed in this article are solely those of the authors and do not necessarily represent those of their affiliated organizations, or those of the publisher, the editors and the reviewers. Any product that may be evaluated in this article, or claim that may be made by its manufacturer, is not guaranteed or endorsed by the publisher.
