# Supplementary material for: Screening for Asymptomatic Coronary Artery Disease via Exercise Stress Testing in Patients With Type 2 Diabetes Mellitus: A Systematic Review and Meta-Analysis
Source: Front Cardiovasc Med. 2021 Nov 1;8:770648. doi: 10.3389/fcvm.2021.770648 (PMC8591186; doi:10.3389/fcvm.2021.770648)
Supplement: Supplementary file 1 [file Data_Sheet_1.docx]

| **Title** | **Page number** |
| --- | --- |
| **Supplementary PRISMA DTA Checklist** | 2-3 |
| **Supplementary Methods** |  |
| Search strategy- Pubmed | 5 |
| Search strategy- Web of Science | 7 |
| Search strategy- MEDLINE(ovid) | 9 |
| Search strategy- Embase | 11 |
| Search strategy- Scopus | 13 |
| Search strategy- EBSCOhost | 18 |
| Search strategy- Ovid | 21 |
| Search strategy- ClinicalTrial.gov | 23 |
| **Supplementary Results** |  |
| **Supplementary Figure 1.** Methodological quality summary: researchers' judgements about methodological quality items for each included study. | 24 |
| **Supplementary Figure 2.** Methodological quality graph: researchers' judgements on each methodological quality item presented as percentages summarizing all included studies. | 25 |
| **Supplementary Table 1.** Additional data - 2 x 2 table. | 26 |
| **Supplementary Figure 3.** SROC plot of sensitivity and (1-specificity) of included studies for EST. | 27 |
| **Supplementary Figure 4.** Forest plots of included studies estimates of sensitivity (a), specificity (b), positive LR (c), negative LR (d) and diagnostic OR (e) for EST. | 28 |
| **Supplementary Figure 5.** Sensitivity analysis of included studies for EST | 29 |
| **Supplementary Figure 6.** Deek’s funnel plots asymmetry test for EST. | 30 |
| **Supplementary Figure 7.** Frequency of missing data for all variables in table 1 for which some observations were missing. | 31 |

| **Section/topic** | **#** | **PRISMA-DTA Checklist Item** | **Reported on page #** |
| --- | --- | --- | --- |
| **TITLE / ABSTRACT** | | |  |
| Title | 1 | Identify the report as a systematic review (+/- meta-analysis) of diagnostic test accuracy (DTA) studies. | 1 |
| Abstract | 2 | Abstract: See PRISMA-DTA for abstracts. | 1-2 |
| **INTRODUCTION** | | |  |
| Rationale | 3 | Describe the rationale for the review in the context of what is already known. | 2 |
| Clinical role of index test | D1 | State the scientific and clinical background, including the intended use and clinical role of the index test, and if applicable, the rationale for minimally acceptable test accuracy (or minimum difference in accuracy for comparative design). | 2 |
| Objectives | 4 | Provide an explicit statement of question(s) being addressed in terms of participants, index test(s), and target condition(s). | 2 |
| **METHODS** | | |  |
| Protocol and registration | 5 | Indicate if a review protocol exists, if and where it can be accessed (e.g., Web address), and, if available, provide registration information including registration number. | 2 |
| Eligibility criteria | 6 | Specify study characteristics (participants, setting, index test(s), reference standard(s), target condition(s), and study design) and report characteristics (e.g., years considered, language, publication status) used as criteria for eligibility, giving rationale. | 2 |
| Information sources | 7 | Describe all information sources (e.g., databases with dates of coverage, contact with study authors to identify additional studies) in the search and date last searched. | 2-3 |
| Search | 8 | Present full search strategies for all electronic databases and other sources searched, including any limits used, such that they could be repeated. | 2-3 |
| Study selection | 9 | State the process for selecting studies (i.e., screening, eligibility, included in systematic review, and, if applicable, included in the meta-analysis). | 3 |
| Data collection process | 10 | Describe method of data extraction from reports (e.g., piloted forms, independently, in duplicate) and any processes for obtaining and confirming data from investigators. | 3 |
| Definitions for data extraction | 11 | Provide definitions used in data extraction and classifications of target condition(s), index test(s), reference standard(s) and other characteristics (e.g. study design, clinical setting). | 3 |
| Risk of bias and applicability | 12 | Describe methods used for assessing risk of bias in individual studies and concerns regarding the applicability to the review question. | 3 |
| Diagnostic accuracy measures | 13 | State the principal diagnostic accuracy measure(s) reported (e.g. sensitivity, specificity) and state the unit of assessment (e.g. per-patient, per-lesion). | 3 |
| Synthesis of results | 14 | Describe methods of handling data, combining results of studies and describing variability between studies. This could include, but is not limited to: a) handling of multiple definitions of target condition. b) handling of multiple thresholds of test positivity, c) handling multiple index test readers, d) handling of indeterminate test results, e) grouping and comparing tests, f) handling of different reference standards | 3 |

**Supplementary PRISMA DTA Checklist**

| **Section/topic** | **#** | **PRISMA-DTA Checklist Item** | **Reported on page #** |
| --- | --- | --- | --- |
| Meta-analysis | D2 | Report the statistical methods used for meta-analyses, if performed. | 3 |
| Additional analyses | 16 | Describe methods of additional analyses (e.g., sensitivity or subgroup analyses, meta-regression), if done, indicating which were pre-specified. | 3 |
| **RESULTS** | | |  |
| Study selection | 17 | Provide numbers of studies screened, assessed for eligibility, included in the review (and included in meta-analysis, if applicable) with reasons for exclusions at each stage, ideally with a flow diagram. | 3 |
| Study characteristics | 18 | For each included study provide citations and present key characteristics including: a) participant characteristics (presentation, prior testing), b) clinical setting, c) study design, d) target condition definition, e) index test, f) reference standard, g) sample size, h) funding sources | 3 |
| Risk of bias and applicability | 19 | Present evaluation of risk of bias and concerns regarding applicability for each study. | 5 |
| Results of individual studies | 20 | For each analysis in each study (e.g. unique combination of index test, reference standard, and positivity threshold) report 2x2 data (TP, FP, FN, TN) with estimates of diagnostic accuracy and confidence intervals, ideally with a forest or receiver operator characteristic (ROC) plot. | 5-7 |
| Synthesis of results | 21 | Describe test accuracy, including variability; if meta-analysis was done, include results and confidence intervals. | 5-7 |
| Additional analysis | 23 | Give results of additional analyses, if done (e.g., sensitivity or subgroup analyses, meta-regression; analysis of index test: failure rates, proportion of inconclusive results, adverse events). | 7-8 |
| **DISCUSSION** | | |  |
| Summary of evidence | 24 | Summarize the main findings including the strength of evidence. | 8-9 |
| Limitations | 25 | Discuss limitations from included studies (e.g. risk of bias and concerns regarding applicability) and from the review process (e.g. incomplete retrieval of identified research). | 10 |
| Conclusions | 26 | Provide a general interpretation of the results in the context of other evidence. Discuss implications for future research and clinical practice (e.g. the intended use and clinical role of the index test). | 10 |
| **FUNDING** | | |  |
| Funding | 27 | For the systematic review, describe the sources of funding and other support and the role of the funders. | 11 |

*Adapted From:*  McInnes MDF, Moher D, Thombs BD, McGrath TA, Bossuyt PM, The PRISMA-DTA Group (2018). Preferred Reporting Items for a Systematic Review and Meta-analysis of Diagnostic Test Accuracy Studies: The PRISMA-DTA Statement. JAMA. 2018 Jan 23;319(4):388-396. doi: 10.1001/jama.2017.19163.

For more information, visit: **www.prisma-statement.org**.

**Supplementary Methods**

**Search strategy on PubMed (www.ncbi.nlm.nih.gov) on** **June 8, 2021.**

**(1800 - 2021)**

("Exercise Test"[MeSH Terms] OR ("exercise ecg"[Title/Abstract] OR "exercise electrocardiography"[Title/Abstract] OR "stress ecg"[Title/Abstract] OR "ecg stress"[Title/Abstract] OR "treadmill ecg"[Title/Abstract] OR "exercise tests"[Title/Abstract] OR "test exercise"[Title/Abstract] OR "tests exercise"[Title/Abstract] OR "exercise testing"[Title/Abstract] OR "testing exercise"[Title/Abstract] OR "eurofit test battery"[Title/Abstract] OR ("eurofit"[All Fields] AND "test batteries"[Title/Abstract]) OR "test battery eurofit"[Title/Abstract] OR "eurofit tests"[Title/Abstract] OR "eurofit test"[Title/Abstract] OR "test eurofit"[Title/Abstract] OR "tests eurofit"[Title/Abstract] OR (("european"[All Fields] OR "europeans"[All Fields]) AND "fitness testing battery"[Title/Abstract]) OR "arm ergometry test"[Title/Abstract] OR "arm ergometry tests"[Title/Abstract] OR (("Ergometry"[MeSH Terms] OR "Ergometry"[All Fields] OR "ergometries"[All Fields]) AND "test arm"[Title/Abstract]) OR (("Ergometry"[MeSH Terms] OR "Ergometry"[All Fields] OR "ergometries"[All Fields]) AND "tests arm"[Title/Abstract]) OR (("research design"[MeSH Terms] OR ("research"[All Fields] AND "design"[All Fields]) OR "research design"[All Fields] OR "Test"[All Fields]) AND "arm ergometry"[Title/Abstract]) OR "tests arm ergometry"[Title/Abstract] OR "fitness testing"[Title/Abstract] OR (("Fitness"[All Fields] OR "fitnesses"[All Fields]) AND "Testings"[Title/Abstract]) OR "testing fitness"[Title/Abstract] OR "cardiopulmonary exercise test"[Title/Abstract] OR "cardiopulmonary exercise tests"[Title/Abstract] OR "exercise test cardiopulmonary"[Title/Abstract] OR "exercise tests cardiopulmonary"[Title/Abstract] OR "test cardiopulmonary exercise"[Title/Abstract] OR "tests cardiopulmonary exercise"[Title/Abstract] OR "cardiopulmonary exercise testing"[Title/Abstract] OR "exercise testing cardiopulmonary"[Title/Abstract] OR "testing cardiopulmonary exercise"[Title/Abstract] OR "step test"[Title/Abstract] OR "step tests"[Title/Abstract] OR "test step"[Title/Abstract] OR "tests step"[Title/Abstract] OR "stress test"[Title/Abstract] OR "stress tests"[Title/Abstract] OR "test stress"[Title/Abstract] OR "tests stress"[Title/Abstract] OR "treadmill test"[Title/Abstract] OR "test treadmill"[Title/Abstract] OR "tests treadmill"[Title/Abstract] OR "treadmill tests"[Title/Abstract] OR "physical fitness testing"[Title/Abstract] OR (("Fitness"[All Fields] OR "fitnesses"[All Fields]) AND "testing physical"[Title/Abstract]) OR "testing physical fitness"[Title/Abstract] OR "bicycle ergometry test"[Title/Abstract] OR "bicycle ergometry tests"[Title/Abstract] OR (("Ergometry"[MeSH Terms] OR "Ergometry"[All Fields] OR "ergometries"[All Fields]) AND "test bicycle"[Title/Abstract]) OR (("Ergometry"[MeSH Terms] OR "Ergometry"[All Fields] OR "ergometries"[All Fields]) AND "tests bicycle"[Title/Abstract]) OR "test bicycle ergometry"[Title/Abstract] OR "tests bicycle ergometry"[Title/Abstract])) AND ("diabetes mellitus, type 2"[MeSH Terms] OR ("diabetes mellitus noninsulin dependent"[Title/Abstract] OR (("diabetes mellitus"[MeSH Terms] OR ("Diabetes"[All Fields] AND "Mellitus"[All Fields]) OR "diabetes mellitus"[All Fields]) AND "Ketosis-Resistant"[Title/Abstract]) OR (("diabetes mellitus"[MeSH Terms] OR ("Diabetes"[All Fields] AND "Mellitus"[All Fields]) OR "diabetes mellitus"[All Fields]) AND "Ketosis-Resistant"[Title/Abstract]) OR "ketosis resistant diabetes mellitus"[Title/Abstract] OR "diabetes mellitus non insulin dependent"[Title/Abstract] OR "diabetes mellitus non insulin dependent"[Title/Abstract] OR "non insulin dependent diabetes mellitus"[Title/Abstract] OR "diabetes mellitus stable"[Title/Abstract] OR "stable diabetes mellitus"[Title/Abstract] OR "diabetes mellitus type ii"[Title/Abstract] OR "NIDDM"[Title/Abstract] OR "diabetes mellitus noninsulin dependent"[Title/Abstract] OR "diabetes mellitus maturity onset"[Title/Abstract] OR "diabetes mellitus maturity onset"[Title/Abstract] OR "maturity onset diabetes mellitus"[Title/Abstract] OR "maturity onset diabetes mellitus"[Title/Abstract] OR "MODY"[Title/Abstract] OR "diabetes mellitus slow onset"[Title/Abstract] OR "diabetes mellitus slow onset"[Title/Abstract] OR ("Slow-Onset"[All Fields] AND "diabetes mellitus"[Title/Abstract]) OR "type 2 diabetes mellitus"[Title/Abstract] OR "noninsulin dependent diabetes mellitus"[Title/Abstract] OR "noninsulin dependent diabetes mellitus"[Title/Abstract] OR "maturity onset diabetes"[Title/Abstract] OR "diabetes maturity onset"[Title/Abstract] OR "maturity onset diabetes"[Title/Abstract] OR "type 2 diabetes"[Title/Abstract] OR "diabetes type 2"[Title/Abstract] OR "diabetes mellitus adult onset"[Title/Abstract] OR "adult onset diabetes mellitus"[Title/Abstract] OR "diabetes mellitus adult onset"[Title/Abstract])) AND ("Coronary Artery Disease"[MeSH Terms] OR "Myocardial Ischemia"[MeSH Terms] OR "Heart Diseases"[MeSH Terms] OR ("artery disease coronary"[Title/Abstract] OR "artery diseases coronary"[Title/Abstract] OR "coronary artery diseases"[Title/Abstract] OR "left main coronary artery disease"[Title/Abstract] OR "left main disease"[Title/Abstract] OR "left main diseases"[Title/Abstract] OR "left main coronary disease"[Title/Abstract] OR "coronary arteriosclerosis"[Title/Abstract] OR (("Arteriosclerosis"[MeSH Terms] OR "Arteriosclerosis"[All Fields] OR "Arterioscleroses"[All Fields]) AND "Coronary"[Title/Abstract]) OR (("coronaries"[All Fields] OR "Heart"[MeSH Terms] OR "Heart"[All Fields] OR "Coronary"[All Fields]) AND "Arterioscleroses"[Title/Abstract]) OR "atherosclerosis coronary"[Title/Abstract] OR (("Atherosclerosis"[MeSH Terms] OR "Atherosclerosis"[All Fields] OR "Atheroscleroses"[All Fields]) AND "Coronary"[Title/Abstract]) OR (("coronaries"[All Fields] OR "Heart"[MeSH Terms] OR "Heart"[All Fields] OR "Coronary"[All Fields]) AND "Atheroscleroses"[Title/Abstract]) OR "coronary atherosclerosis"[Title/Abstract] OR "arteriosclerosis coronary"[Title/Abstract] OR "ischemia myocardial"[Title/Abstract] OR "ischemias myocardial"[Title/Abstract] OR "myocardial ischemias"[Title/Abstract] OR "ischemic heart disease"[Title/Abstract] OR "heart disease ischemic"[Title/Abstract] OR "disease ischemic heart"[Title/Abstract] OR "diseases ischemic heart"[Title/Abstract] OR "heart diseases ischemic"[Title/Abstract] OR "ischemic heart diseases"[Title/Abstract] OR "heart disease"[Title/Abstract] OR "cardiac diseases"[Title/Abstract] OR "cardiac disease"[Title/Abstract] OR "cardiac disorders"[Title/Abstract] OR "cardiac disorder"[Title/Abstract] OR "heart disorders"[Title/Abstract] OR "heart disorder"[Title/Abstract])) AND ("sensitiv*"[Title/Abstract] OR "sensitivity and specificity"[MeSH Terms] OR ("predictive"[Title/Abstract] AND "value*"[Title/Abstract]) OR "predictive value of tests"[MeSH Terms] OR "accuracy*"[Title/Abstract])

**Search strategy on Web of Science on June 8, 2021**

**(1950-2021)**

| # | Results | Search |
| --- | --- | --- |
| # 12 | [860](http://libdb.csu.edu.cn/summary.do?product=UA&doc=1&qid=29&SID=6DC3akgqbytKZezteNZ&search_mode=CombineSearches&update_back2search_link_param=yes) | #11 AND #10 AND #6 AND #3 |
| # 11 | [4,506,641](http://libdb.csu.edu.cn/summary.do?product=UA&doc=1&qid=27&SID=6DC3akgqbytKZezteNZ&search_mode=GeneralSearch&update_back2search_link_param=yes) | **Topic:** (sensitiv* OR (sensitivity and specificity)  OR (predictive AND value*) OR predictive value of tests OR accuracy*) |
| # 10 | [2,031,741](http://libdb.csu.edu.cn/summary.do?product=UA&doc=1&qid=26&SID=6DC3akgqbytKZezteNZ&search_mode=CombineSearches&update_back2search_link_param=yes) | #9 OR #8 OR #7 |
| # 9 | [1,888,988](http://libdb.csu.edu.cn/summary.do?product=UA&doc=1&qid=25&SID=6DC3akgqbytKZezteNZ&search_mode=GeneralSearch&update_back2search_link_param=yes) | **Topic:** (Heart Diseases OR Heart Disease OR Cardiac Diseases OR Cardiac Disease OR Cardiac Disorders OR Cardiac Disorder OR Heart Disorders OR Heart Disorder) |
| # 8 | [602,206](http://libdb.csu.edu.cn/summary.do?product=UA&doc=1&qid=24&SID=6DC3akgqbytKZezteNZ&search_mode=GeneralSearch&update_back2search_link_param=yes) | **Topic:** (Artery Disease, Coronary OR Artery Diseases, Coronary OR Coronary Artery Diseases OR Left Main Coronary Artery Disease OR Left Main Disease OR Left Main Diseases OR Left Main Coronary Disease OR Coronary Arteriosclerosis OR Arterioscleroses, Coronary OR Coronary Arterioscleroses OR Atherosclerosis, Coronary OR Atheroscleroses, Coronary OR Coronary Atheroscleroses OR Coronary Atherosclerosis OR Arteriosclerosis, Coronary OR Ischemia, Myocardial OR Ischemias, Myocardial OR Myocardial Ischemias OR Ischemic Heart Disease OR Heart Disease, Ischemic OR Disease, Ischemic Heart OR Diseases, Ischemic Heart OR Heart Diseases, Ischemic OR Ischemic Heart Diseases) |
| # 7 | [451,333](http://libdb.csu.edu.cn/summary.do?product=UA&doc=1&qid=23&SID=6DC3akgqbytKZezteNZ&search_mode=GeneralSearch&update_back2search_link_param=yes) | **Topic:** (Coronary Artery Disease OR Myocardial Ischemia) |
| # 6 | [383,130](http://libdb.csu.edu.cn/summary.do?product=UA&doc=1&qid=22&SID=6DC3akgqbytKZezteNZ&search_mode=CombineSearches&update_back2search_link_param=yes) | #5 OR #4 |
| # 5 | [383,130](http://libdb.csu.edu.cn/summary.do?product=UA&doc=1&qid=21&SID=6DC3akgqbytKZezteNZ&search_mode=GeneralSearch&update_back2search_link_param=yes) | **Topic:** (Diabetes Mellitus, Noninsulin-Dependent OR Diabetes Mellitus, Ketosis-Resistant OR Diabetes Mellitus, Ketosis Resistant OR Ketosis-Resistant Diabetes Mellitus OR Diabetes Mellitus, Non Insulin Dependent OR Diabetes Mellitus, Non-Insulin-Dependent OR Non-Insulin-Dependent Diabetes Mellitus OR Diabetes Mellitus, Stable OR Stable Diabetes Mellitus OR Diabetes Mellitus, Type II OR NIDDM OR Diabetes Mellitus, Noninsulin Dependent OR Diabetes Mellitus, Maturity-Onset OR Diabetes Mellitus, Maturity Onset OR Maturity-Onset Diabetes Mellitus OR Maturity Onset Diabetes Mellitus OR MODY OR Diabetes Mellitus, Slow-Onset OR Diabetes Mellitus, Slow Onset OR Slow-Onset Diabetes Mellitus OR Type 2 Diabetes Mellitus OR Noninsulin-Dependent Diabetes Mellitus OR Noninsulin Dependent Diabetes Mellitus OR Maturity-Onset Diabetes OR Diabetes, Maturity-Onset OR Maturity Onset Diabetes OR Type 2 Diabetes OR Diabetes, Type 2 OR Diabetes Mellitus, Adult-Onset OR Adult-Onset Diabetes Mellitus OR Diabetes Mellitus, Adult Onset) |
| # 4 | [306,078](http://libdb.csu.edu.cn/summary.do?product=UA&doc=1&qid=20&SID=6DC3akgqbytKZezteNZ&search_mode=GeneralSearch&update_back2search_link_param=yes) | **Topic:** (Diabetes Mellitus, Type 2) |
| # 3 | [1,006,806](http://libdb.csu.edu.cn/summary.do?product=UA&doc=1&qid=19&SID=6DC3akgqbytKZezteNZ&search_mode=CombineSearches&update_back2search_link_param=yes) | #2 OR #1 |
| # 2 | [1,006,806](http://libdb.csu.edu.cn/summary.do?product=UA&doc=1&qid=18&SID=6DC3akgqbytKZezteNZ&search_mode=GeneralSearch&update_back2search_link_param=yes) | **Topic:** (Exercise ECG OR Exercise Electrocardiography OR Stress ECG OR ECG Stress OR Treadmill ECG OR Exercise Tests OR Test, Exercise OR Tests, Exercise OR Exercise Testing OR Testing, Exercise OR Eurofit Test Battery OR Eurofit Test Batteries OR Test Battery, Eurofit OR EuroFit Tests OR EuroFit Test OR Test, EuroFit OR Tests, EuroFit OR European Fitness Testing Battery OR Arm Ergometry Test OR Arm Ergometry Tests OR Ergometry Test, Arm OR Ergometry Tests, Arm OR Test, Arm Ergometry OR Tests, Arm Ergometry OR Fitness Testing OR Fitness Testings OR Testing, Fitness OR Cardiopulmonary Exercise Test OR Cardiopulmonary Exercise Tests OR Exercise Test, Cardiopulmonary OR Exercise Tests, Cardiopulmonary OR Test, Cardiopulmonary Exercise OR Tests, Cardiopulmonary Exercise OR Cardiopulmonary Exercise Testing OR Exercise Testing, Cardiopulmonary OR Testing, Cardiopulmonary Exercise OR Step Test OR Step Tests OR Test, Step OR Tests, Step OR Stress Test OR Stress Tests OR Test, Stress OR Tests, Stress OR Treadmill Test OR Test, Treadmill OR Tests, Treadmill OR Treadmill Tests OR Physical Fitness Testing OR Fitness Testing, Physical OR Testing, Physical Fitness OR Bicycle Ergometry Test OR Bicycle Ergometry Tests OR Ergometry Test, Bicycle OR Ergometry Tests, Bicycle OR Test, Bicycle Ergometry OR Tests, Bicycle Ergometry) |
| # 1 | [229,645](http://libdb.csu.edu.cn/summary.do?product=UA&doc=1&qid=17&SID=6DC3akgqbytKZezteNZ&search_mode=GeneralSearch&update_back2search_link_param=yes) | **Topic:** (Exercise Test) |

**Search strategy on MEDLINE(ovid) on June 8, 2021**

**(1946 to June 07, 2021)**

| # | Search | Results |
| --- | --- | --- |
| 1 | exp Exercise Test/ | 66803 |
| 2 | (Exercise Tests or Test, Exercise or Tests, Exercise or Exercise Testing or Testing, Exercise or Eurofit Test Battery or Eurofit Test Batteries or Test Battery, Eurofit or EuroFit Tests or EuroFit Test or Test, EuroFit or Tests, EuroFit or European Fitness Testing Battery or Arm Ergometry Test or Arm Ergometry Tests or Ergometry Test, Arm or Ergometry Tests, Arm or Test, Arm Ergometry or Tests, Arm Ergometry or Fitness Testing or Fitness Testings or Testing, Fitness or Cardiopulmonary Exercise Test or Cardiopulmonary Exercise Tests or Exercise Test, Cardiopulmonary or Exercise Tests, Cardiopulmonary or Test, Cardiopulmonary Exercise or Tests, Cardiopulmonary Exercise or Cardiopulmonary Exercise Testing or Exercise Testing, Cardiopulmonary or Testing, Cardiopulmonary Exercise or Step Test or Step Tests or Test, Step or Tests, Step or Stress Test or Stress Tests or Test, Stress or Tests, Stress or Treadmill Test or Test, Treadmill or Tests, Treadmill or Treadmill Tests or Physical Fitness Testing or Fitness Testing, Physical or Testing, Physical Fitness or Bicycle Ergometry Test or Bicycle Ergometry Tests or Ergometry Test, Bicycle or Ergometry Tests, Bicycle or Test, Bicycle Ergometry or Tests, Bicycle Ergometry).af. | 33343 |
| 3 | (Exercise ECG or Exercise Electrocardiography or Stress ECG or ECG Stress or Treadmill ECG).af. | 2174 |
| 4 | 1 or 2 or 3 | 84586 |
| 5 | exp Diabetes Mellitus, Type 2/ | 141878 |
| 6 | (Diabetes Mellitus, Noninsulin-Dependent or Diabetes Mellitus, Ketosis-Resistant or Diabetes Mellitus, Ketosis Resistant or Ketosis-Resistant Diabetes Mellitus or Diabetes Mellitus, Non Insulin Dependent or Diabetes Mellitus, Non-Insulin-Dependent or Non-Insulin-Dependent Diabetes Mellitus or Diabetes Mellitus, Stable or Stable Diabetes Mellitus or Diabetes Mellitus, Type II or NIDDM or Diabetes Mellitus, Noninsulin Dependent or Diabetes Mellitus, Maturity-Onset or Diabetes Mellitus, Maturity Onset or Maturity-Onset Diabetes Mellitus or Maturity Onset Diabetes Mellitus or MODY or Diabetes Mellitus, Slow-Onset or Diabetes Mellitus, Slow Onset or Slow-Onset Diabetes Mellitus or Type 2 Diabetes Mellitus or Noninsulin-Dependent Diabetes Mellitus or Noninsulin Dependent Diabetes Mellitus or Maturity-Onset Diabetes or Diabetes, Maturity-Onset or Maturity Onset Diabetes or Type 2 Diabetes or Diabetes, Type 2 or Diabetes Mellitus, Adult-Onset or Adult-Onset Diabetes Mellitus or Diabetes Mellitus, Adult Onset).af. | 153985 |
| 7 | 5 or 6 | 201422 |
| 8 | exp Coronary Artery Disease/ | 65877 |
| 9 | exp Myocardial Ischemia/ | 441776 |
| 10 | exp Heart Diseases/ | 1165930 |
| 11 | (Artery Disease, Coronary or Artery Diseases, Coronary or Coronary Artery Diseases or Left Main Coronary Artery Disease or Left Main Disease or Left Main Diseases or Left Main Coronary Disease or Coronary Arteriosclerosis or Arterioscleroses, Coronary or Coronary Arterioscleroses or Atherosclerosis, Coronary or Atheroscleroses, Coronary or Coronary Atheroscleroses or Coronary Atherosclerosis or Arteriosclerosis, Coronary or Ischemia, Myocardial or Ischemias, Myocardial or Myocardial Ischemias or Ischemic Heart Disease or Heart Disease, Ischemic or Disease, Ischemic Heart or Diseases, Ischemic Heart or Heart Diseases, Ischemic or Ischemic Heart Diseases).af. | 40789 |
| 12 | (Heart Disease or Cardiac Diseases or Cardiac Disease or Cardiac Disorders or Cardiac Disorder or Heart Disorders or Heart Disorder).af. | 212107 |
| 13 | 8 or 9 or 10 or 11 or 12 | 1239751 |
| 14 | exp "Predictive Value of Tests"/ | 211330 |
| 15 | (sensitiv* or (predictive and value*) or accuracy*).af. | 2291290 |
| 16 | (sensitivity and specificity).mp. | 513228 |
| 17 | 14 or 15 or 16 | 2291291 |
| 18 | 4 and 7 and 13 and 17 | 99 |

**Search strategy on Embase on June 9, 2021**

Embase <1974 to 2021 June 08>

| **#** | **Query** | **Results from 9 Jun 2021** |
| --- | --- | --- |
| 1 | exercise test/ | 59,302 |
| 2 | (Exercise Tests or Test, Exercise or Tests, Exercise or Exercise Testing or Testing, Exercise or Eurofit Test Battery or Eurofit Test Batteries or Test Battery, Eurofit or EuroFit Tests or EuroFit Test or Test, EuroFit or Tests, EuroFit or European Fitness Testing Battery or Arm Ergometry Test or Arm Ergometry Tests or Ergometry Test, Arm or Ergometry Tests, Arm or Test, Arm Ergometry or Tests, Arm Ergometry or Fitness Testing or Fitness Testings or Testing, Fitness or Cardiopulmonary Exercise Test or Cardiopulmonary Exercise Tests or Exercise Test, Cardiopulmonary or Exercise Tests, Cardiopulmonary or Test, Cardiopulmonary Exercise or Tests, Cardiopulmonary Exercise or Cardiopulmonary Exercise Testing or Exercise Testing, Cardiopulmonary or Testing, Cardiopulmonary Exercise or Step Test or Step Tests or Test, Step or Tests, Step or Stress Test or Stress Tests or Test, Stress or Tests, Stress or Treadmill Test or Test, Treadmill or Tests, Treadmill or Treadmill Tests or Physical Fitness Testing or Fitness Testing, Physical or Testing, Physical Fitness or Bicycle Ergometry Test or Bicycle Ergometry Tests or Ergometry Test, Bicycle or Ergometry Tests, Bicycle or Test, Bicycle Ergometry or Tests, Bicycle Ergometry).ab,kw,ot,ti. | 51,839 |
| 3 | 1 or 2 | 86,475 |
| 4 | non insulin dependent diabetes mellitus/ | 276,497 |
| 5 | (Diabetes Mellitus, Noninsulin-Dependent or Diabetes Mellitus, Ketosis-Resistant or Diabetes Mellitus, Ketosis Resistant or Ketosis-Resistant Diabetes Mellitus or Diabetes Mellitus, Non Insulin Dependent or Diabetes Mellitus, Non-Insulin-Dependent or Non-Insulin-Dependent Diabetes Mellitus or Diabetes Mellitus, Stable or Stable Diabetes Mellitus or Diabetes Mellitus, Type II or NIDDM or Diabetes Mellitus, Noninsulin Dependent or Diabetes Mellitus, Maturity-Onset or Diabetes Mellitus, Maturity Onset or Maturity-Onset Diabetes Mellitus or Maturity Onset Diabetes Mellitus or MODY or Diabetes Mellitus, Slow-Onset or Diabetes Mellitus, Slow Onset or Slow-Onset Diabetes Mellitus or Type 2 Diabetes Mellitus or Noninsulin-Dependent Diabetes Mellitus or Noninsulin Dependent Diabetes Mellitus or Maturity-Onset Diabetes or Diabetes, Maturity-Onset or Maturity Onset Diabetes or Type 2 Diabetes or Diabetes, Type 2 or Diabetes Mellitus, Adult-Onset or Adult-Onset Diabetes Mellitus or Diabetes Mellitus, Adult Onset).ab,kw,ot,ti. | 231,699 |
| 6 | 4 or 5 | 322,769 |
| 7 | coronary artery disease/ | 200,990 |
| 8 | ischemic heart disease/ | 130,797 |
| 9 | heart disease/ | 112,604 |
| 10 | (Artery Disease, Coronary or Artery Diseases, Coronary or Coronary Artery Diseases or Left Main Coronary Artery Disease or Left Main Disease or Left Main Diseases or Left Main Coronary Disease or Coronary Arteriosclerosis or Arterioscleroses, Coronary or Coronary Arterioscleroses or Atherosclerosis, Coronary or Atheroscleroses, Coronary or Coronary Atheroscleroses or Coronary Atherosclerosis or Arteriosclerosis, Coronary or Ischemia, Myocardial or Ischemias, Myocardial or Myocardial Ischemias or Ischemic Heart Disease or Heart Disease, Ischemic or Disease, Ischemic Heart or Diseases, Ischemic Heart or Heart Diseases, Ischemic or Ischemic Heart Diseases, Heart Disease or Cardiac Diseases or Cardiac Disease or Cardiac Disorders or Cardiac Disorder or Heart Disorders or Heart Disorder).ab,kw,ot,ti. | 94,153 |
| 11 | 7 or 8 or 9 or 10 | 468,742 |
| 12 | (sensitiv: or predictive value:).mp. or accurac:.tw. | 2,741,484 |
| 13 | 3 and 6 and 11 and 12 | 64 |

**Search strategy on Scopus on June 9, 2021**

| History Count | Search Terms | Results |
| --- | --- | --- |
| 13 | ( ( TITLE-ABS-KEY ( "Exercise Test" ) )  OR  ( ( "Exercise Tests" )  OR  ( "Test, Exercise" )  OR  ( "Tests, Exercise" )  OR  ( "Exercise Testing" )  OR  ( "Testing, Exercise" )  OR  ( "Eurofit Test Battery" )  OR  ( "Eurofit Test Batteries" )  OR  ( "Test Battery, Eurofit" )  OR  ( "EuroFit Tests" )  OR  ( "EuroFit Test" )  OR  ( "Test, EuroFit" )  OR  ( "Tests, EuroFit" )  OR  ( "European Fitness Testing Battery" )  OR  ( "Arm Ergometry Test" )  OR  ( "Arm Ergometry Tests" )  OR  ( "Ergometry Test, Arm" )  OR  ( "Ergometry Tests, Arm" )  OR  ( "Test, Arm Ergometry" )  OR  ( "Tests, Arm Ergometry" )  OR  ( "Fitness Testing" )  OR  ( "Fitness Testings" )  OR  ( "Testing, Fitness" )  OR  ( "Cardiopulmonary Exercise Test" )  OR  ( "Cardiopulmonary Exercise Tests" )  OR  ( "Exercise Test, Cardiopulmonary" )  OR  ( "Exercise Tests, Cardiopulmonary" )  OR  ( "Test, Cardiopulmonary Exercise" )  OR  ( "Tests, Cardiopulmonary Exercise" )  OR  ( "Cardiopulmonary Exercise Testing" )  OR  ( "Exercise Testing, Cardiopulmonary" )  OR  ( "Testing, Cardiopulmonary Exercise" )  OR  ( "Step Test" )  OR  ( "Step Tests" )  OR  ( "Test, Step" )  OR  ( "Tests, Step" )  OR  ( "Stress Test" )  OR  ( "Stress Tests" )  OR  ( "Test, Stress" )  OR  ( "Tests, Stress" )  OR  ( "Treadmill Test" )  OR  ( "Test, Treadmill" )  OR  ( "Tests, Treadmill" )  OR  ( "Treadmill Tests" )  OR  ( "Physical Fitness Testing" )  OR  ( "Fitness Testing, Physical" )  OR  ( "Testing, Physical Fitness" )  OR  ( "Bicycle Ergometry Test" )  OR  ( "Bicycle Ergometry Tests" )  OR  ( "Ergometry Test, Bicycle" )  OR  ( "Ergometry Tests, Bicycle" )  OR  ( "Test, Bicycle Ergometry" )  OR  ( "Tests, Bicycle Ergometry" )  OR  ( "Exercise ECG" )  OR  ( "exercise electrocardiography" )  OR  ( "stress ecg" )  OR  ( "ecg stress" )  OR  ( "treadmill ecg" ) ) )  AND  ( ( TITLE-ABS-KEY ( "Diabetes Mellitus, Type 2" ) )  OR  ( ( "Diabetes Mellitus, Noninsulin-Dependent" )  OR  ( "Diabetes Mellitus, Ketosis-Resistant" )  OR  ( "Diabetes Mellitus, Ketosis Resistant" )  OR  ( "Ketosis-Resistant Diabetes Mellitus" )  OR  ( "Diabetes Mellitus, Non Insulin Dependent" )  OR  ( "Diabetes Mellitus, Non-Insulin-Dependent" )  OR  ( "Non-Insulin-Dependent Diabetes Mellitus" )  OR  ( "Diabetes Mellitus, Stable" )  OR  ( "Stable Diabetes Mellitus" )  OR  ( "Diabetes Mellitus, Type II" )  OR  ( "NIDDM" )  OR  ( "Diabetes Mellitus, Noninsulin Dependent" )  OR  ( "Diabetes Mellitus, Maturity-Onset" )  OR  ( "Diabetes Mellitus, Maturity Onset" )  OR  ( "Maturity-Onset Diabetes Mellitus" )  OR  ( "Maturity Onset Diabetes Mellitus" )  OR  ( "MODY" )  OR  ( "Diabetes Mellitus, Slow-Onset" )  OR  ( "Diabetes Mellitus, Slow Onset" )  OR  ( "Slow-Onset Diabetes Mellitus" )  OR  ( "Type 2 Diabetes Mellitus" )  OR  ( "Noninsulin-Dependent Diabetes Mellitus" )  OR  ( "Noninsulin Dependent Diabetes Mellitus" )  OR  ( "Maturity-Onset Diabetes" )  OR  ( "Diabetes, Maturity-Onset" )  OR  ( "Maturity Onset Diabetes" )  OR  ( "Type 2 Diabetes" )  OR  ( "Diabetes, Type 2" )  OR  ( "Diabetes Mellitus, Adult-Onset" )  OR  ( "Adult-Onset Diabetes Mellitus" )  OR  ( "Diabetes Mellitus, Adult Onset" ) ) )  AND  ( ( TITLE-ABS-KEY ( "Myocardial Ischemia" ) )  OR  ( TITLE-ABS-KEY ( "Heart disease" ) )  OR  ( TITLE-ABS-KEY ( "Coronary Artery Disease" ) )  OR  ( ( "Artery Disease, Coronary" )  OR  ( "Artery Diseases, Coronary" )  OR  ( "Coronary Artery Diseases" )  OR  ( "Left Main Coronary Artery Disease" )  OR  ( "Left Main Disease" )  OR  ( "Left Main Diseases" )  OR  ( "Left Main Coronary Disease" )  OR  ( "Coronary Arteriosclerosis" )  OR  ( "Arterioscleroses, Coronary" )  OR  ( "Coronary Arterioscleroses" )  OR  ( "Atherosclerosis, Coronary" )  OR  ( "Atheroscleroses, Coronary" )  OR  ( "Coronary Atheroscleroses" )  OR  ( "Coronary Atherosclerosis" )  OR  ( "Arteriosclerosis, Coronary" )  OR  ( "Coronary Diseases" )  OR  ( "Disease, Coronary" )  OR  ( "Diseases, Coronary" )  OR  ( "Coronary Heart Disease" )  OR  ( "Coronary Heart Diseases" )  OR  ( "Disease, Coronary Heart" )  OR  ( "Diseases, Coronary Heart" )  OR  ( "Heart Disease, Coronary" )  OR  ( "Heart Diseases, Coronary" )  OR  ( "Heart Disease" )  OR  ( "Cardiac Diseases" )  OR  ( "Cardiac Disease" )  OR  ( "Cardiac Disorders" )  OR  ( "Cardiac Disorder" )  OR  ( "Heart Disorders" )  OR  ( "Heart Disorder" )  OR  ( "Ischemia, Myocardial" )  OR  ( "Ischemias, Myocardial" )  OR  ( "Myocardial Ischemias" )  OR  ( "Ischemic Heart Disease" )  OR  ( "Heart Disease, Ischemic" )  OR  ( "Disease, Ischemic Heart" )  OR  ( "Diseases, Ischemic Heart" )  OR  ( "Heart Diseases, Ischemic" )  OR  ( "Ischemic Heart Diseases" ) ) )  AND  ( TITLE-ABS-KEY ( ( sensitiv*  OR  ( sensitivity  AND  specificity )  OR  ( predictive  AND  value* )  OR  predictive  AND  value  AND  of  AND  tests  OR  accuracy* ) ) ) | [679](http://libdb.csu.edu.cn/search/history/results.uri?origin=searchhistory&shid=20) |
| 12 | TITLE-ABS-KEY ( ( sensitiv*  OR  ( sensitivity  AND  specificity )  OR  ( predictive  AND  value* )  OR  predictive  AND  value  AND  of  AND  tests  OR  accuracy* ) ) | [454,967](http://libdb.csu.edu.cn/search/history/results.uri?origin=searchhistory&shid=19) |
| 11 | ( TITLE-ABS-KEY ( "Myocardial Ischemia" ) )  OR  ( TITLE-ABS-KEY ( "Heart disease" ) )  OR  ( TITLE-ABS-KEY ( "Coronary Artery Disease" ) )  OR  ( ( "Artery Disease, Coronary" )  OR  ( "Artery Diseases, Coronary" )  OR  ( "Coronary Artery Diseases" )  OR  ( "Left Main Coronary Artery Disease" )  OR  ( "Left Main Disease" )  OR  ( "Left Main Diseases" )  OR  ( "Left Main Coronary Disease" )  OR  ( "Coronary Arteriosclerosis" )  OR  ( "Arterioscleroses, Coronary" )  OR  ( "Coronary Arterioscleroses" )  OR  ( "Atherosclerosis, Coronary" )  OR  ( "Atheroscleroses, Coronary" )  OR  ( "Coronary Atheroscleroses" )  OR  ( "Coronary Atherosclerosis" )  OR  ( "Arteriosclerosis, Coronary" )  OR  ( "Coronary Diseases" )  OR  ( "Disease, Coronary" )  OR  ( "Diseases, Coronary" )  OR  ( "Coronary Heart Disease" )  OR  ( "Coronary Heart Diseases" )  OR  ( "Disease, Coronary Heart" )  OR  ( "Diseases, Coronary Heart" )  OR  ( "Heart Disease, Coronary" )  OR  ( "Heart Diseases, Coronary" )  OR  ( "Heart Disease" )  OR  ( "Cardiac Diseases" )  OR  ( "Cardiac Disease" )  OR  ( "Cardiac Disorders" )  OR  ( "Cardiac Disorder" )  OR  ( "Heart Disorders" )  OR  ( "Heart Disorder" )  OR  ( "Ischemia, Myocardial" )  OR  ( "Ischemias, Myocardial" )  OR  ( "Myocardial Ischemias" )  OR  ( "Ischemic Heart Disease" )  OR  ( "Heart Disease, Ischemic" )  OR  ( "Disease, Ischemic Heart" )  OR  ( "Diseases, Ischemic Heart" )  OR  ( "Heart Diseases, Ischemic" )  OR  ( "Ischemic Heart Diseases" ) ) | [1,575,564](http://libdb.csu.edu.cn/search/history/results.uri?origin=searchhistory&shid=13) |
| 10 | ( "Artery Disease, Coronary" )  OR  ( "Artery Diseases, Coronary" )  OR  ( "Coronary Artery Diseases" )  OR  ( "Left Main Coronary Artery Disease" )  OR  ( "Left Main Disease" )  OR  ( "Left Main Diseases" )  OR  ( "Left Main Coronary Disease" )  OR  ( "Coronary Arteriosclerosis" )  OR  ( "Arterioscleroses, Coronary" )  OR  ( "Coronary Arterioscleroses" )  OR  ( "Atherosclerosis, Coronary" )  OR  ( "Atheroscleroses, Coronary" )  OR  ( "Coronary Atheroscleroses" )  OR  ( "Coronary Atherosclerosis" )  OR  ( "Arteriosclerosis, Coronary" )  OR  ( "Coronary Diseases" )  OR  ( "Disease, Coronary" )  OR  ( "Diseases, Coronary" )  OR  ( "Coronary Heart Disease" )  OR  ( "Coronary Heart Diseases" )  OR  ( "Disease, Coronary Heart" )  OR  ( "Diseases, Coronary Heart" )  OR  ( "Heart Disease, Coronary" )  OR  ( "Heart Diseases, Coronary" )  OR  ( "Heart Disease" )  OR  ( "Cardiac Diseases" )  OR  ( "Cardiac Disease" )  OR  ( "Cardiac Disorders" )  OR  ( "Cardiac Disorder" )  OR  ( "Heart Disorders" )  OR  ( "Heart Disorder" )  OR  ( "Ischemia, Myocardial" )  OR  ( "Ischemias, Myocardial" )  OR  ( "Myocardial Ischemias" )  OR  ( "Ischemic Heart Disease" )  OR  ( "Heart Disease, Ischemic" )  OR  ( "Disease, Ischemic Heart" )  OR  ( "Diseases, Ischemic Heart" )  OR  ( "Heart Diseases, Ischemic" )  OR  ( "Ischemic Heart Diseases" ) | [1,552,871](http://libdb.csu.edu.cn/search/history/results.uri?origin=searchhistory&shid=12) |
| 9 | TITLE-ABS-KEY ( "Coronary Artery Disease" ) | [242,228](http://libdb.csu.edu.cn/search/history/results.uri?origin=searchhistory&shid=11) |
| 8 | TITLE-ABS-KEY ( "Heart disease" ) | [421,376](http://libdb.csu.edu.cn/search/history/results.uri?origin=searchhistory&shid=10) |
| 7 | TITLE-ABS-KEY ( "Myocardial Ischemia" ) | [63,045](http://libdb.csu.edu.cn/search/history/results.uri?origin=searchhistory&shid=9) |
| 6 | ( TITLE-ABS-KEY ( "Diabetes Mellitus, Type 2" ) )  OR  ( ( "Diabetes Mellitus, Noninsulin-Dependent" )  OR  ( "Diabetes Mellitus, Ketosis-Resistant" )  OR  ( "Diabetes Mellitus, Ketosis Resistant" )  OR  ( "Ketosis-Resistant Diabetes Mellitus" )  OR  ( "Diabetes Mellitus, Non Insulin Dependent" )  OR  ( "Diabetes Mellitus, Non-Insulin-Dependent" )  OR  ( "Non-Insulin-Dependent Diabetes Mellitus" )  OR  ( "Diabetes Mellitus, Stable" )  OR  ( "Stable Diabetes Mellitus" )  OR  ( "Diabetes Mellitus, Type II" )  OR  ( "NIDDM" )  OR  ( "Diabetes Mellitus, Noninsulin Dependent" )  OR  ( "Diabetes Mellitus, Maturity-Onset" )  OR  ( "Diabetes Mellitus, Maturity Onset" )  OR  ( "Maturity-Onset Diabetes Mellitus" )  OR  ( "Maturity Onset Diabetes Mellitus" )  OR  ( "MODY" )  OR  ( "Diabetes Mellitus, Slow-Onset" )  OR  ( "Diabetes Mellitus, Slow Onset" )  OR  ( "Slow-Onset Diabetes Mellitus" )  OR  ( "Type 2 Diabetes Mellitus" )  OR  ( "Noninsulin-Dependent Diabetes Mellitus" )  OR  ( "Noninsulin Dependent Diabetes Mellitus" )  OR  ( "Maturity-Onset Diabetes" )  OR  ( "Diabetes, Maturity-Onset" )  OR  ( "Maturity Onset Diabetes" )  OR  ( "Type 2 Diabetes" )  OR  ( "Diabetes, Type 2" )  OR  ( "Diabetes Mellitus, Adult-Onset" )  OR  ( "Adult-Onset Diabetes Mellitus" )  OR  ( "Diabetes Mellitus, Adult Onset" ) ) | [875,454](http://libdb.csu.edu.cn/search/history/results.uri?origin=searchhistory&shid=7) |
| 5 | ( "Diabetes Mellitus, Noninsulin-Dependent" )  OR  ( "Diabetes Mellitus, Ketosis-Resistant" )  OR  ( "Diabetes Mellitus, Ketosis Resistant" )  OR  ( "Ketosis-Resistant Diabetes Mellitus" )  OR  ( "Diabetes Mellitus, Non Insulin Dependent" )  OR  ( "Diabetes Mellitus, Non-Insulin-Dependent" )  OR  ( "Non-Insulin-Dependent Diabetes Mellitus" )  OR  ( "Diabetes Mellitus, Stable" )  OR  ( "Stable Diabetes Mellitus" )  OR  ( "Diabetes Mellitus, Type II" )  OR  ( "NIDDM" )  OR  ( "Diabetes Mellitus, Noninsulin Dependent" )  OR  ( "Diabetes Mellitus, Maturity-Onset" )  OR  ( "Diabetes Mellitus, Maturity Onset" )  OR  ( "Maturity-Onset Diabetes Mellitus" )  OR  ( "Maturity Onset Diabetes Mellitus" )  OR  ( "MODY" )  OR  ( "Diabetes Mellitus, Slow-Onset" )  OR  ( "Diabetes Mellitus, Slow Onset" )  OR  ( "Slow-Onset Diabetes Mellitus" )  OR  ( "Type 2 Diabetes Mellitus" )  OR  ( "Noninsulin-Dependent Diabetes Mellitus" )  OR  ( "Noninsulin Dependent Diabetes Mellitus" )  OR  ( "Maturity-Onset Diabetes" )  OR  ( "Diabetes, Maturity-Onset" )  OR  ( "Maturity Onset Diabetes" )  OR  ( "Type 2 Diabetes" )  OR  ( "Diabetes, Type 2" )  OR  ( "Diabetes Mellitus, Adult-Onset" )  OR  ( "Adult-Onset Diabetes Mellitus" )  OR  ( "Diabetes Mellitus, Adult Onset" ) | [871,424](http://libdb.csu.edu.cn/search/history/results.uri?origin=searchhistory&shid=6) |
| 4 | TITLE-ABS-KEY ( "Diabetes Mellitus, Type 2" ) | [121,292](http://libdb.csu.edu.cn/search/history/results.uri?origin=searchhistory&shid=4) |
| 3 | ( TITLE-ABS-KEY ( "Exercise Test" ) )  OR  ( ( "Exercise Tests" )  OR  ( "Test, Exercise" )  OR  ( "Tests, Exercise" )  OR  ( "Exercise Testing" )  OR  ( "Testing, Exercise" )  OR  ( "Eurofit Test Battery" )  OR  ( "Eurofit Test Batteries" )  OR  ( "Test Battery, Eurofit" )  OR  ( "EuroFit Tests" )  OR  ( "EuroFit Test" )  OR  ( "Test, EuroFit" )  OR  ( "Tests, EuroFit" )  OR  ( "European Fitness Testing Battery" )  OR  ( "Arm Ergometry Test" )  OR  ( "Arm Ergometry Tests" )  OR  ( "Ergometry Test, Arm" )  OR  ( "Ergometry Tests, Arm" )  OR  ( "Test, Arm Ergometry" )  OR  ( "Tests, Arm Ergometry" )  OR  ( "Fitness Testing" )  OR  ( "Fitness Testings" )  OR  ( "Testing, Fitness" )  OR  ( "Cardiopulmonary Exercise Test" )  OR  ( "Cardiopulmonary Exercise Tests" )  OR  ( "Exercise Test, Cardiopulmonary" )  OR  ( "Exercise Tests, Cardiopulmonary" )  OR  ( "Test, Cardiopulmonary Exercise" )  OR  ( "Tests, Cardiopulmonary Exercise" )  OR  ( "Cardiopulmonary Exercise Testing" )  OR  ( "Exercise Testing, Cardiopulmonary" )  OR  ( "Testing, Cardiopulmonary Exercise" )  OR  ( "Step Test" )  OR  ( "Step Tests" )  OR  ( "Test, Step" )  OR  ( "Tests, Step" )  OR  ( "Stress Test" )  OR  ( "Stress Tests" )  OR  ( "Test, Stress" )  OR  ( "Tests, Stress" )  OR  ( "Treadmill Test" )  OR  ( "Test, Treadmill" )  OR  ( "Tests, Treadmill" )  OR  ( "Treadmill Tests" )  OR  ( "Physical Fitness Testing" )  OR  ( "Fitness Testing, Physical" )  OR  ( "Testing, Physical Fitness" )  OR  ( "Bicycle Ergometry Test" )  OR  ( "Bicycle Ergometry Tests" )  OR  ( "Ergometry Test, Bicycle" )  OR  ( "Ergometry Tests, Bicycle" )  OR  ( "Test, Bicycle Ergometry" )  OR  ( "Tests, Bicycle Ergometry" )  OR  ( "Exercise ECG" )  OR  ( "exercise electrocardiography" )  OR  ( "stress ecg" )  OR  ( "ecg stress" )  OR  ( "treadmill ecg" ) ) | [194,306](http://libdb.csu.edu.cn/search/history/results.uri?origin=searchhistory&shid=3) |
| 2 | ( "Exercise Tests" )  OR  ( "Test, Exercise" )  OR  ( "Tests, Exercise" )  OR  ( "Exercise Testing" )  OR  ( "Testing, Exercise" )  OR  ( "Eurofit Test Battery" )  OR  ( "Eurofit Test Batteries" )  OR  ( "Test Battery, Eurofit" )  OR  ( "EuroFit Tests" )  OR  ( "EuroFit Test" )  OR  ( "Test, EuroFit" )  OR  ( "Tests, EuroFit" )  OR  ( "European Fitness Testing Battery" )  OR  ( "Arm Ergometry Test" )  OR  ( "Arm Ergometry Tests" )  OR  ( "Ergometry Test, Arm" )  OR  ( "Ergometry Tests, Arm" )  OR  ( "Test, Arm Ergometry" )  OR  ( "Tests, Arm Ergometry" )  OR  ( "Fitness Testing" )  OR  ( "Fitness Testings" )  OR  ( "Testing, Fitness" )  OR  ( "Cardiopulmonary Exercise Test" )  OR  ( "Cardiopulmonary Exercise Tests" )  OR  ( "Exercise Test, Cardiopulmonary" )  OR  ( "Exercise Tests, Cardiopulmonary" )  OR  ( "Test, Cardiopulmonary Exercise" )  OR  ( "Tests, Cardiopulmonary Exercise" )  OR  ( "Cardiopulmonary Exercise Testing" )  OR  ( "Exercise Testing, Cardiopulmonary" )  OR  ( "Testing, Cardiopulmonary Exercise" )  OR  ( "Step Test" )  OR  ( "Step Tests" )  OR  ( "Test, Step" )  OR  ( "Tests, Step" )  OR  ( "Stress Test" )  OR  ( "Stress Tests" )  OR  ( "Test, Stress" )  OR  ( "Tests, Stress" )  OR  ( "Treadmill Test" )  OR  ( "Test, Treadmill" )  OR  ( "Tests, Treadmill" )  OR  ( "Treadmill Tests" )  OR  ( "Physical Fitness Testing" )  OR  ( "Fitness Testing, Physical" )  OR  ( "Testing, Physical Fitness" )  OR  ( "Bicycle Ergometry Test" )  OR  ( "Bicycle Ergometry Tests" )  OR  ( "Ergometry Test, Bicycle" )  OR  ( "Ergometry Tests, Bicycle" )  OR  ( "Test, Bicycle Ergometry" )  OR  ( "Tests, Bicycle Ergometry" )  OR  ( "Exercise ECG" )  OR  ( "exercise electrocardiography" )  OR  ( "stress ecg" )  OR  ( "ecg stress" )  OR  ( "treadmill ecg" ) | [194,306](http://libdb.csu.edu.cn/search/history/results.uri?origin=searchhistory&shid=2) |
| 1 | TITLE-ABS-KEY ( "Exercise Test" ) | [86,085](http://libdb.csu.edu.cn/search/history/results.uri?origin=searchhistory&shid=1) |

**Search strategy on EBSCOhost on June 9, 2021**

| **#** | **Search Terms** | **Results** |
| --- | --- | --- |
| S13 | (SU ( sensitiv* OR sensitivity and specificity[MeSH Terms] OR (predictive AND value*) OR predictive value of tests[MeSH Term] OR accuracy* )) AND (S3 AND S6 AND S11 AND S12) | 22 |
| S12 | SU sensitiv* OR sensitivity and specificity[MeSH Terms] OR (predictive AND value*) OR predictive value of tests[MeSH Term] OR accuracy* | 802,935 |
| S11 | (SU (Artery Disease, Coronary) OR (Artery Diseases, Coronary) OR (Coronary Artery Diseases) OR (Left Main Coronary Artery Disease) OR (Left Main Disease) OR (Left Main Diseases) OR (Left Main Coronary Disease) OR (Coronary Arteriosclerosis) OR (Arterioscleroses, Coronary) OR (Coronary Arterioscleroses) OR (Atherosclerosis, Coronary) OR (Atheroscleroses, Coronary) OR (Coronary Atheroscleroses) OR (Coronary Atherosclerosis) OR (Arteriosclerosis, Coronary) OR (Coronary Diseases) OR (Disease, Coronary) OR (Diseases, Coronary) OR (Coronary Heart Disease) OR (Coronary Heart Diseases) OR (Disease, Coronary Heart) OR (Diseases, Coronary Heart) OR (Heart Disease, Coronary) OR (Heart Diseases, Coronary) OR (Heart Disease) OR (Cardiac Diseases) OR (Cardiac Disease) OR (Cardiac Disorders) OR (Cardiac Disorder) OR (Heart Disorders) OR (Heart Disorder) OR (Ischemia, Myocardial) OR (Ischemias, Myocardial) OR (Myocardial Ischemias) OR (Ischemic Heart Disease) OR (Heart Disease, Ischemic) OR (Disease, Ischemic Heart) OR (Diseases, Ischemic Heart) OR (Heart Diseases, Ischemic) OR (Ischemic Heart Diseases)) AND (S7 OR S8 OR S9 OR S10) | 504,180 |
| S10 | SU (Artery Disease, Coronary) OR (Artery Diseases, Coronary) OR (Coronary Artery Diseases) OR (Left Main Coronary Artery Disease) OR (Left Main Disease) OR (Left Main Diseases) OR (Left Main Coronary Disease) OR (Coronary Arteriosclerosis) OR (Arterioscleroses, Coronary) OR (Coronary Arterioscleroses) OR (Atherosclerosis, Coronary) OR (Atheroscleroses, Coronary) OR (Coronary Atheroscleroses) OR (Coronary Atherosclerosis) OR (Arteriosclerosis, Coronary) OR (Coronary Diseases) OR (Disease, Coronary) OR (Diseases, Coronary) OR (Coronary Heart Disease) OR (Coronary Heart Diseases) OR (Disease, Coronary Heart) OR (Diseases, Coronary Heart) OR (Heart Disease, Coronary) OR (Heart Diseases, Coronary) OR (Heart Disease) OR (Cardiac Diseases) OR (Cardiac Disease) OR (Cardiac Disorders) OR (Cardiac Disorder) OR (Heart Disorders) OR (Heart Disorder) OR (Ischemia, Myocardial) OR (Ischemias, Myocardial) OR (Myocardial Ischemias) OR (Ischemic Heart Disease) OR (Heart Disease, Ischemic) OR (Disease, Ischemic Heart) OR (Diseases, Ischemic Heart) OR (Heart Diseases, Ischemic) OR (Ischemic Heart Diseases) | 504,180 |
| S9 | SU Heart disease | 226,690 |
| S8 | SU Myocardial Ischemia | 41,149 |
| S7 | SU Coronary Artery Disease | 72,460 |
| S6 | (SU (Diabetes Mellitus, Noninsulin-Dependent) OR (Diabetes Mellitus, Ketosis-Resistant) OR (Diabetes Mellitus, Ketosis Resistant) OR (Ketosis-Resistant Diabetes Mellitus) OR (Diabetes Mellitus, Non Insulin Dependent) OR (Diabetes Mellitus, Non-Insulin-Dependent) OR (Non-Insulin-Dependent Diabetes Mellitus) OR (Diabetes Mellitus, Stable) OR (Stable Diabetes Mellitus) OR (Diabetes Mellitus, Type II) OR (NIDDM) OR (Diabetes Mellitus, Noninsulin Dependent) OR (Diabetes Mellitus, Maturity-Onset) OR (Diabetes Mellitus, Maturity Onset) OR (Maturity-Onset Diabetes Mellitus) OR (Maturity Onset Diabetes Mellitus) OR (MODY) OR (Diabetes Mellitus, Slow-Onset) OR (Diabetes Mellitus, Slow Onset) OR (Slow-Onset Diabetes Mellitus) OR (Type 2 Diabetes Mellitus) OR (Noninsulin-Dependent Diabetes Mellitus) OR (Noninsulin Dependent Diabetes Mellitus) OR (Maturity-Onset Diabetes) OR (Diabetes, Maturity-Onset) OR (Maturity Onset Diabetes) OR (Type 2 Diabetes) OR (Diabetes, Type 2) OR (Diabetes Mellitus, Adult-Onset) OR (Adult-Onset Diabetes Mellitus) OR (Diabetes Mellitus, Adult Onset)) AND (S4 OR S5) | 219,245 |
| S5 | SU (Diabetes Mellitus, Noninsulin-Dependent) OR (Diabetes Mellitus, Ketosis-Resistant) OR (Diabetes Mellitus, Ketosis Resistant) OR (Ketosis-Resistant Diabetes Mellitus) OR (Diabetes Mellitus, Non Insulin Dependent) OR (Diabetes Mellitus, Non-Insulin-Dependent) OR (Non-Insulin-Dependent Diabetes Mellitus) OR (Diabetes Mellitus, Stable) OR (Stable Diabetes Mellitus) OR (Diabetes Mellitus, Type II) OR (NIDDM) OR (Diabetes Mellitus, Noninsulin Dependent) OR (Diabetes Mellitus, Maturity-Onset) OR (Diabetes Mellitus, Maturity Onset) OR (Maturity-Onset Diabetes Mellitus) OR (Maturity Onset Diabetes Mellitus) OR (MODY) OR (Diabetes Mellitus, Slow-Onset) OR (Diabetes Mellitus, Slow Onset) OR (Slow-Onset Diabetes Mellitus) OR (Type 2 Diabetes Mellitus) OR (Noninsulin-Dependent Diabetes Mellitus) OR (Noninsulin Dependent Diabetes Mellitus) OR (Maturity-Onset Diabetes) OR (Diabetes, Maturity-Onset) OR (Maturity Onset Diabetes) OR (Type 2 Diabetes) OR (Diabetes, Type 2) OR (Diabetes Mellitus, Adult-Onset) OR (Adult-Onset Diabetes Mellitus) OR (Diabetes Mellitus, Adult Onset) | 219,245 |
| S4 | SU Diabetes Mellitus, Type 2 | 144,899 |
| S3 | (SU (Exercise ECG) OR (exercise electrocardiography) OR (stress ecg) OR (ecg stress) OR (treadmill ecg) OR (Exercise Tests) OR (Test, Exercise) OR (Tests, Exercise) OR (Exercise Testing) OR (Testing, Exercise) OR (Eurofit Test Battery) OR (Eurofit Test Batteries) OR (Test Battery, Eurofit) OR (EuroFit Tests) OR (EuroFit Test) OR (Test, EuroFit) OR (Tests, EuroFit) OR (European Fitness Testing Battery) OR (Arm Ergometry Test) OR (Arm Ergometry Tests) OR (Ergometry Test, Arm) OR (Ergometry Tests, Arm) OR (Test, Arm Ergometry) OR (Tests, Arm Ergometry) OR (Fitness Testing) OR (Fitness Testings) OR (Testing, Fitness) OR (Cardiopulmonary Exercise Test) OR (Cardiopulmonary Exercise Tests) OR (Exercise Test, Cardiopulmonary) OR (Exercise Tests, Cardiopulmonary) OR (Test, Cardiopulmonary Exercise) OR (Tests, Cardiopulmonary Exercise) OR (Cardiopulmonary Exercise Testing) OR (Exercise Testing, Cardiopulmonary) OR (Testing, Cardiopulmonary Exercise) OR (Step Test) OR (Step Tests) OR (Test, Step) OR (Tests, Step) OR (Stress Test) OR (Stress Tests) OR (Test, Stress) OR (Tests, Stress) OR (Treadmill Test) OR (Test, Treadmill) OR (Tests, Treadmill) OR (Treadmill Tests) OR (Physical Fitness Testing) OR (Fitness Testing, Physical) OR (Testing, Physical Fitness) OR (Bicycle Ergometry Test) OR (Bicycle Ergometry Tests) OR (Ergometry Test, Bicycle) OR (Ergometry Tests, Bicycle) OR (Test, Bicycle Ergometry) OR (Tests, Bicycle Ergometry)) AND (S1 OR S2) | 83,497 |
| S2 | SU (Exercise ECG) OR (exercise electrocardiography) OR (stress ecg) OR (ecg stress) OR (treadmill ecg) OR (Exercise Tests) OR (Test, Exercise) OR (Tests, Exercise) OR (Exercise Testing) OR (Testing, Exercise) OR (Eurofit Test Battery) OR (Eurofit Test Batteries) OR (Test Battery, Eurofit) OR (EuroFit Tests) OR (EuroFit Test) OR (Test, EuroFit) OR (Tests, EuroFit) OR (European Fitness Testing Battery) OR (Arm Ergometry Test) OR (Arm Ergometry Tests) OR (Ergometry Test, Arm) OR (Ergometry Tests, Arm) OR (Test, Arm Ergometry) OR (Tests, Arm Ergometry) OR (Fitness Testing) OR (Fitness Testings) OR (Testing, Fitness) OR (Cardiopulmonary Exercise Test) OR (Cardiopulmonary Exercise Tests) OR (Exercise Test, Cardiopulmonary) OR (Exercise Tests, Cardiopulmonary) OR (Test, Cardiopulmonary Exercise) OR (Tests, Cardiopulmonary Exercise) OR (Cardiopulmonary Exercise Testing) OR (Exercise Testing, Cardiopulmonary) OR (Testing, Cardiopulmonary Exercise) OR (Step Test) OR (Step Tests) OR (Test, Step) OR (Tests, Step) OR (Stress Test) OR (Stress Tests) OR (Test, Stress) OR (Tests, Stress) OR (Treadmill Test) OR (Test, Treadmill) OR (Tests, Treadmill) OR (Treadmill Tests) OR (Physical Fitness Testing) OR (Fitness Testing, Physical) OR (Testing, Physical Fitness) OR (Bicycle Ergometry Test) OR (Bicycle Ergometry Tests) OR (Ergometry Test, Bicycle) OR (Ergometry Tests, Bicycle) OR (Test, Bicycle Ergometry) OR (Tests, Bicycle Ergometry) | 83,497 |
| S1 | SU Exercise Test | 78,626 |

**Search strategy on Ovid on June 9, 2021**

| **#** | **Query** | **Results from 9 Jun 2021** |
| --- | --- | --- |
| 1 | exercise test/ | 73,507 |
| 2 | (Exercise ECG or exercise electrocardiography or stress ecg or ecg stress or treadmill ecg or Exercise Tests or Test, Exercise or Tests, Exercise or Exercise Testing or Testing, Exercise or Eurofit Test Battery or Eurofit Test Batteries or Test Battery, Eurofit or EuroFit Tests or EuroFit Test or Test, EuroFit or Tests, EuroFit or European Fitness Testing Battery or Arm Ergometry Test or Arm Ergometry Tests or Ergometry Test, Arm or Ergometry Tests, Arm or Test, Arm Ergometry or Tests, Arm Ergometry or Fitness Testing or Fitness Testings or Testing, Fitness or Cardiopulmonary Exercise Test or Cardiopulmonary Exercise Tests or Exercise Test, Cardiopulmonary or Exercise Tests, Cardiopulmonary or Test, Cardiopulmonary Exercise or Tests, Cardiopulmonary Exercise or Cardiopulmonary Exercise Testing or Exercise Testing, Cardiopulmonary or Testing, Cardiopulmonary Exercise or Step Test or Step Tests or Test, Step or Tests, Step or Stress Test or Stress Tests or Test, Stress or Tests, Stress or Treadmill Test or Test, Treadmill or Tests, Treadmill or Treadmill Tests or Physical Fitness Testing or Fitness Testing, Physical or Testing, Physical Fitness or Bicycle Ergometry Test or Bicycle Ergometry Tests or Ergometry Test, Bicycle or Ergometry Tests, Bicycle or Test, Bicycle Ergometry).ab,hw,kw,ot,sh,ti. | 59,708 |
| 3 | 1 or 2 | 114,112 |
| 4 | diabetes mellitus, type 2/ | 159,653 |
| 5 | (Diabetes Mellitus, Noninsulin-Dependent or Diabetes Mellitus, Ketosis-Resistant or Diabetes Mellitus, Ketosis Resistant or Ketosis-Resistant Diabetes Mellitus or Diabetes Mellitus, Non Insulin Dependent or Diabetes Mellitus, Non-Insulin-Dependent or Non-Insulin-Dependent Diabetes Mellitus or Diabetes Mellitus, Stable or Stable Diabetes Mellitus or Diabetes Mellitus, Type II or NIDDM or Diabetes Mellitus, Noninsulin Dependent or Diabetes Mellitus, Maturity-Onset or Diabetes Mellitus, Maturity Onset or Maturity-Onset Diabetes Mellitus or Maturity Onset Diabetes Mellitus or MODY or Diabetes Mellitus, Slow-Onset or Diabetes Mellitus, Slow Onset or Slow-Onset Diabetes Mellitus or Type 2 Diabetes Mellitus or Noninsulin-Dependent Diabetes Mellitus or Noninsulin Dependent Diabetes Mellitus or Maturity-Onset Diabetes or Diabetes, Maturity-Onset or Maturity Onset Diabetes or Type 2 Diabetes or Diabetes, Type 2 or Diabetes Mellitus, Adult-Onset or Adult-Onset Diabetes Mellitus).ab,hw,kw,ot,sh,ti. | 230,968 |
| 6 | coronary artery disease/ | 72,389 |
| 7 | myocardial ischemia/ | 43,854 |
| 8 | heart diseases/ | 73,493 |
| 9 | (Artery Disease, Coronary or Artery Diseases, Coronary or Coronary Artery Diseases or Left Main Coronary Artery Disease or Left Main Disease or Left Main Diseases or Left Main Coronary Disease or Coronary Arteriosclerosis or Arterioscleroses, Coronary or Coronary Arterioscleroses or Atherosclerosis, Coronary or Atheroscleroses, Coronary or Coronary Atheroscleroses or Coronary Atherosclerosis or Arteriosclerosis, Coronary or Coronary Diseases or Disease, Coronary or Diseases, Coronary or Coronary Heart Disease or Coronary Heart Diseases or Disease, Coronary Heart or Diseases, Coronary Heart or Heart Disease, Coronary or Heart Diseases, Coronary or Heart Disease or Cardiac Diseases or Cardiac Disease or Cardiac Disorders or Cardiac Disorder or Heart Disorders or Heart Disorder or Ischemia, Myocardial or Ischemias, Myocardial or Myocardial Ischemias or Ischemic Heart Disease or Heart Disease, Ischemic or Disease, Ischemic Heart or Diseases, Ischemic Heart or Heart Diseases, Ischemic or Ischemic Heart Diseases).ab,hw,kw,ot,sh,ti. | 319,045 |
| 10 | 4 or 5 | 281,694 |
| 11 | 6 or 7 or 8 or 9 | 460,375 |
| 12 | (sensitiv: or predictive value:).mp. or accurac:.tw. | 4,687,138 |
| 13 | 3 and 10 and 11 and 12 | 86 |

**Search ClinicalTrial.gov on June 9, 2021**

No eligible studies.

**Supplementary Results**


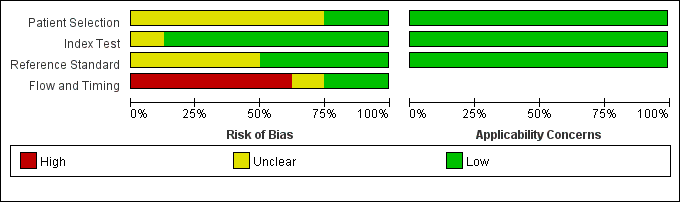


**Supplementary Figure 1.** Methodological quality summary: researchers' judgements about methodological quality items for each included study.


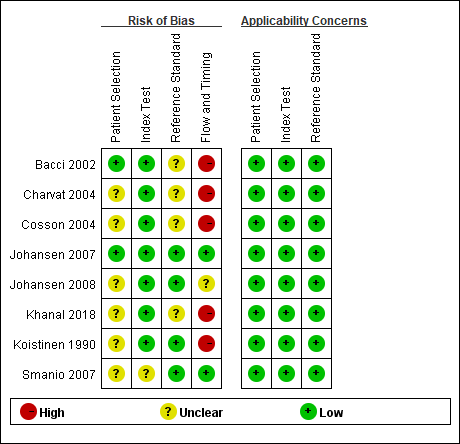


**Supplementary Figure 2.** Methodological quality graph: researchers' judgements on each methodological quality item presented as percentages summarizing all included studies.

**Supplementary Table 1. Additional data - 2x2 table**

| **Author Year** | **Number** | **TP** | **FP** | **FN** | **TN** |
| --- | --- | --- | --- | --- | --- |
| **Koistinen 1990** | NO.1 | 11 | 6 | 2 | 14 |
| **Bacci 2002** | NO.2 | 20 | 7 | 8 | 36 |
| **Charvat 2004** | NO.3 | 15 | 5 | 2 | 8 |
| **Cosson 2004** | NO.4 | 18 | 25 | 16 | 17 |
| **Johansen 2007** | NO.5 | 6 | 18 | 11 | 47 |
| **Smanio 2007a** | NO.6 | 15 | 27 | 19 | 43 |
| **Smanio 2008b** | NO.7 | 15 | 19 | 19 | 51 |
| **Johansen 2008** | NO.8 | 7 | 22 | 16 | 46 |
| **Khanal 2018** | NO.9 | 8 | 11 | 3 | 6 |

TP: True positive; FP: False positive; FN: False negative; TN: True negative.

**
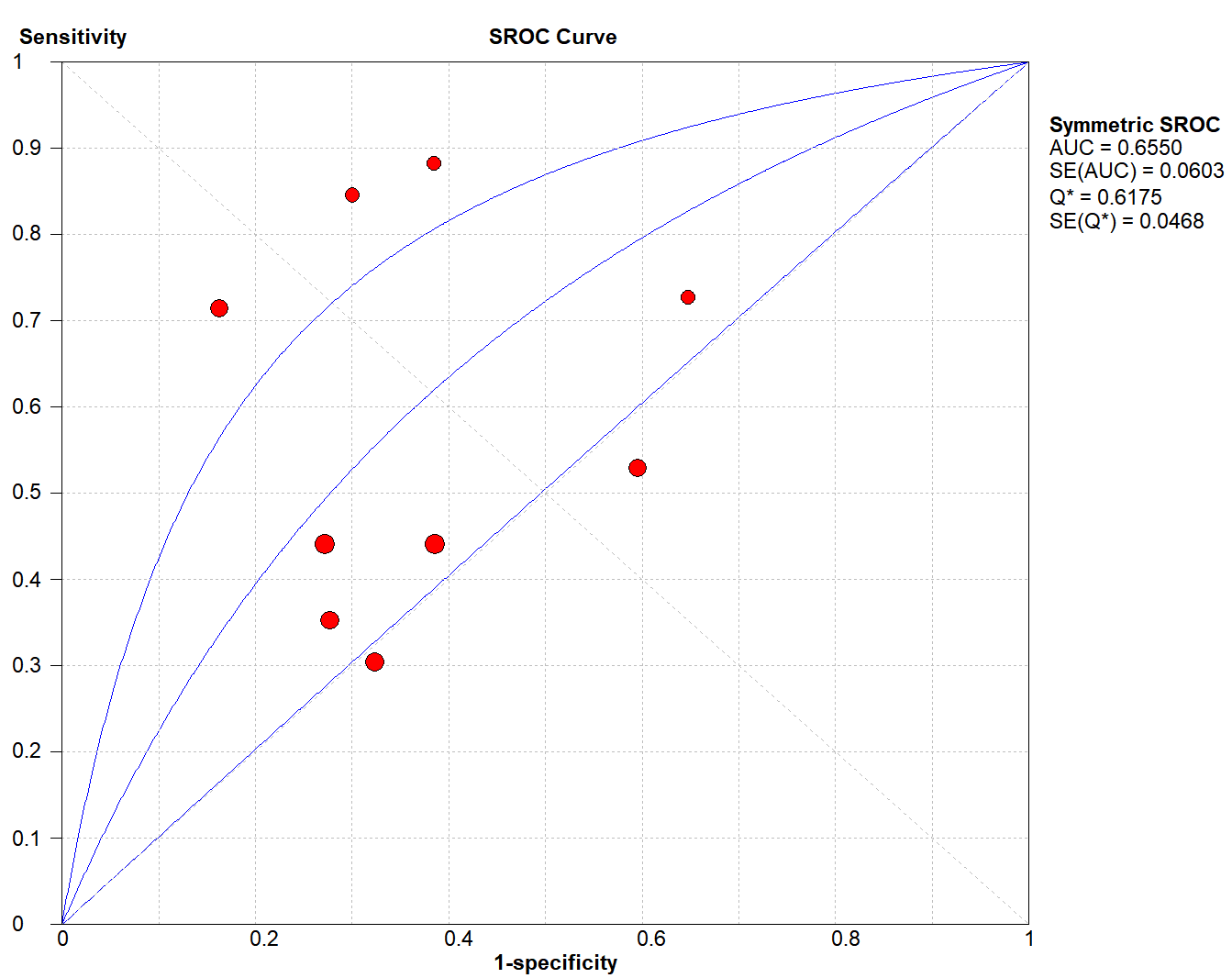
**

**Supplementary Figure 3.** SROC plot of sensitivity and (1-specificity) of included studies for EST.


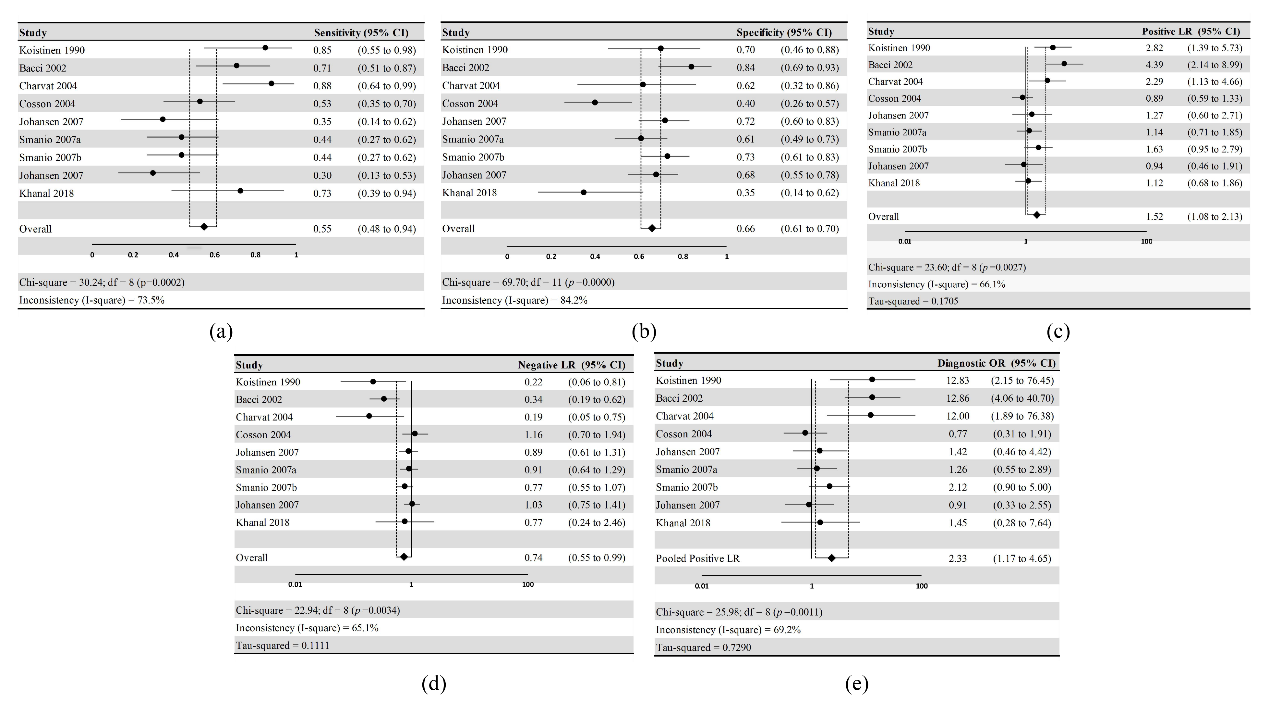


**Supplementary Figure 4.** Forest plot of included studies estimates of sensitivity (a), specificity (b), positive LR (c), negative LR (d) and diagnostic OR (e) for EST.


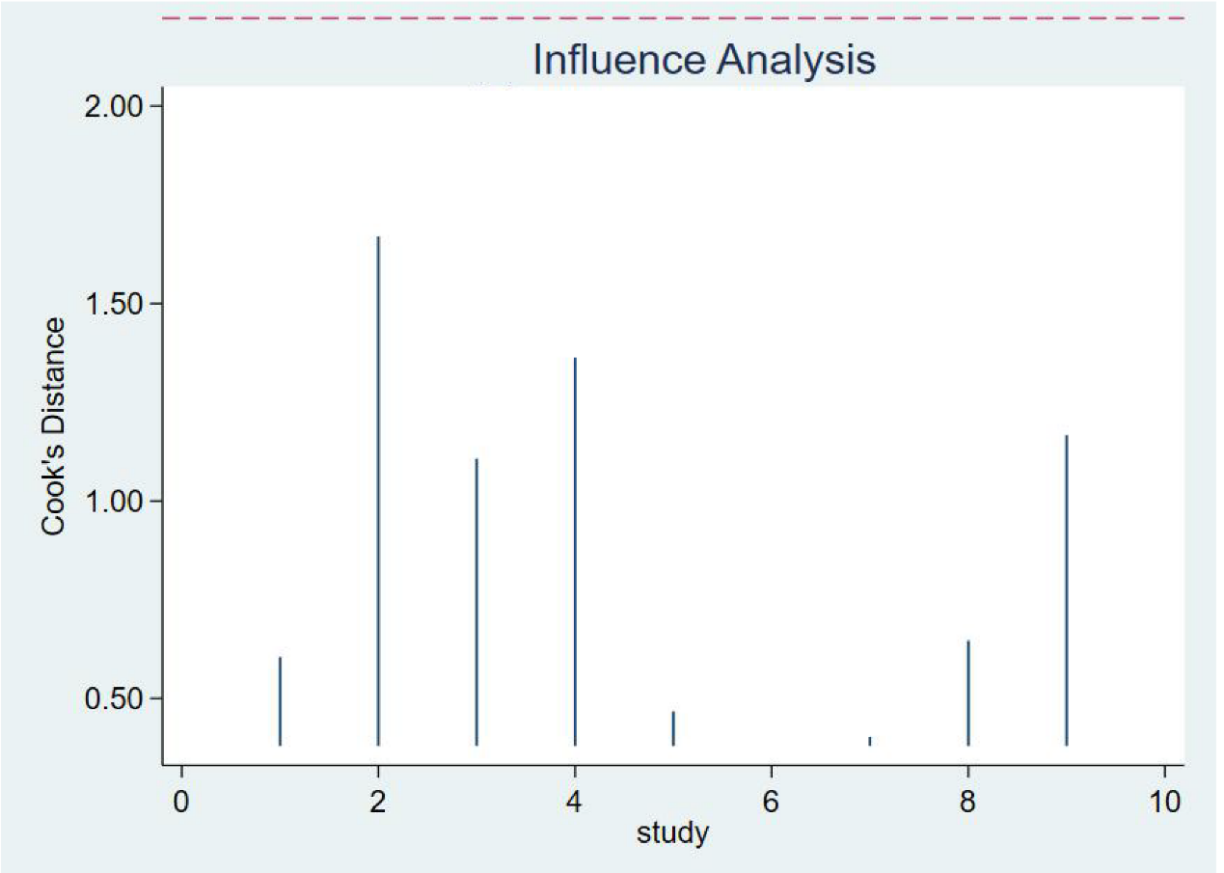


**Supplementary Figure 5.** Sensitivity analysis of included studies for EST.


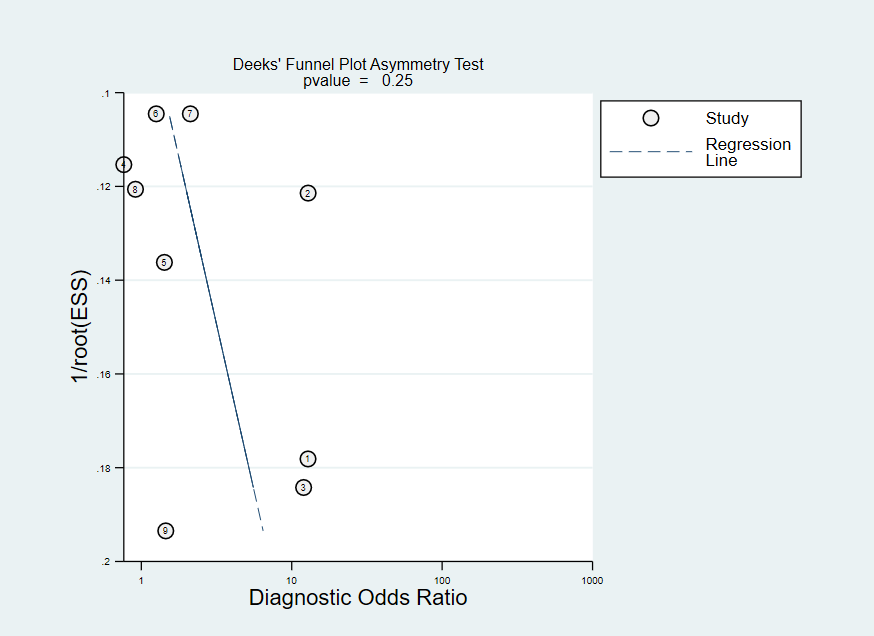


**Supplementary Figure 6.** Deek’s funnel plot asymmetry test for EST


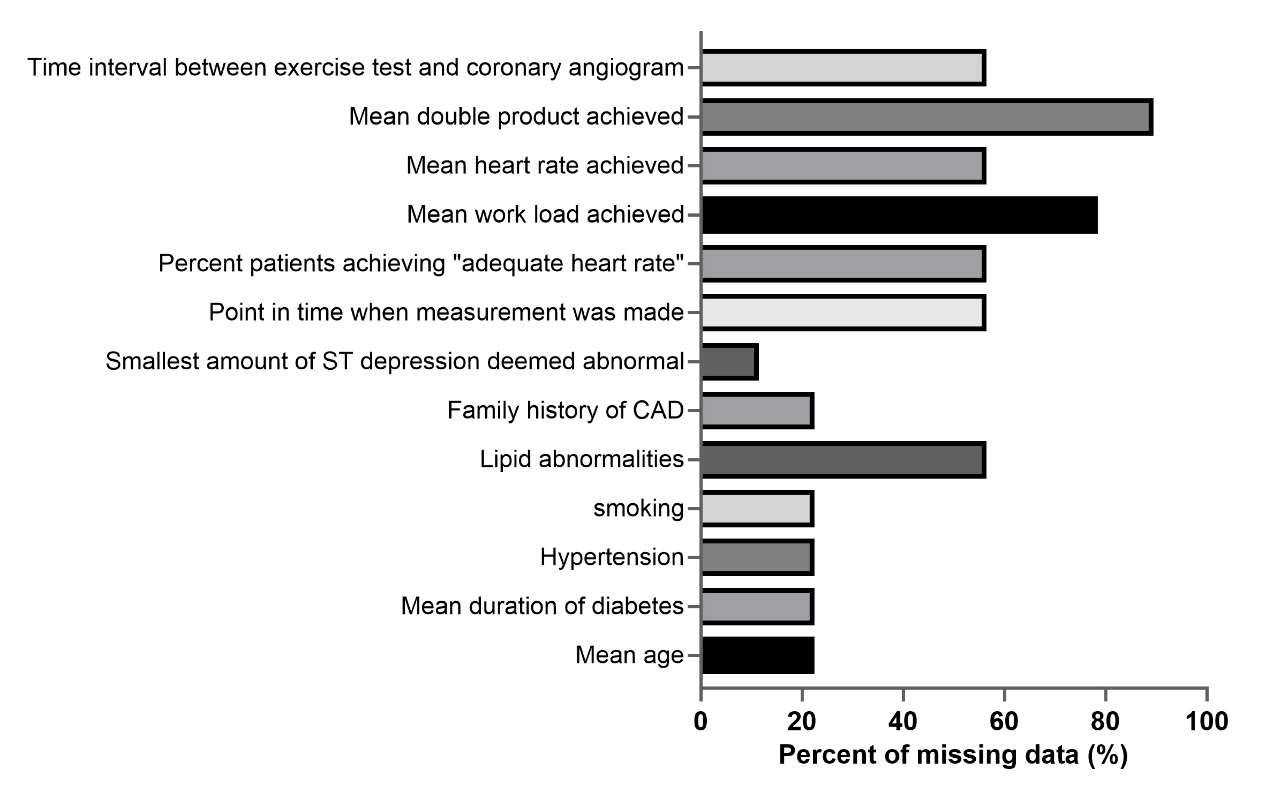


**Supplementary Figure 7.** Frequency of missing data for all variables in table 1 for which some observations were missing.
